# Macrocyclic Peptide Probes for Immunomodulatory Protein CD59: Potent Modulators of Bacterial Toxin Activity and Antibody‐Dependent Cytotoxicity

**DOI:** 10.1002/anie.202422673

**Published:** 2025-05-05

**Authors:** Jasmine K. Bickel, Ammar. I. S. Ahmed, Aidan B. Pidd, Rhodri M. Morgan, Tom E. McAllister, Sam Horrell, Emma C. Couves, Hemavathi Nagaraj, Edward J. Bartlett, Kamel El Omari, Akane Kawamura, Doryen Bubeck, Edward W. Tate

**Affiliations:** ^1^ Department of Chemistry, Molecular Sciences Research Hub Imperial College London London W12 0BZ UK; ^2^ Department of Life Sciences, Sir Ernst Chain Building Imperial College London London SW7 2AZ UK; ^3^ Chemistry–School of Natural and Environmental Sciences Newcastle University Newcastle upon Tyne Newcastle NE1 7RU UK; ^4^ Diamond Light Source Harwell Science & Innovation Campus Didcot Oxford OX11 0DE UK; ^5^ The Francis Crick Institute London NW1 1AT UK

**Keywords:** Cell‐surface receptors, Macrocyclic peptides, Membrane attack complex, Protein‐protein interaction inhibitors, Structure‐guided design

## Abstract

CD59 is an immunomodulatory cell surface receptor associated with human disease. Despite its importance in complement regulation and bacterial pathogenesis, CD59 remains a challenging therapeutic target. Research to date has focused on antibody or protein‐based strategies. Here we present a new approach to target CD59 using macrocyclic peptides with low nanomolar affinity for CD59. Through X‐ray crystallographic studies and structure‐activity relationship (SAR) studies, we identify key interactions that are essential for binding and activity. We find that the macrocyclic peptide CP‐06 adopts a beta‐hairpin structure and binds CD59 through an intermolecular beta‐sheet, mimicking protein–protein interactions of biologically relevant CD59 interaction partners. We create dimeric and lipidated macrocyclic peptide conjugates as enhanced cell‐active CD59 inhibitors and show that these probes can be used to modulate both complement‐mediated killing of human cells and lytic activity of bacterial virulence factors. Together, our data provide a starting point for future development of macrocyclic peptides to target CD59 activity in diverse cellular contexts.

## Graphical abstract

This study presents novel macrocyclic peptides with low nanomolar affinity for CD59, an immunomodulatory cell surface receptor associated with human disease. Crystallographic and structure‐activity relationship (SAR) studies reveal peptide CP‐06 mimics the binding of biologically relevant CD59 interaction partners. Dimeric and lipidated CP‐06 analogues control both complement‐mediated killing of human cells and lytic activity of bacterial virulence factors via CD59 inhibition.

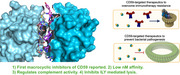

## Introduction

**Figure 1:**
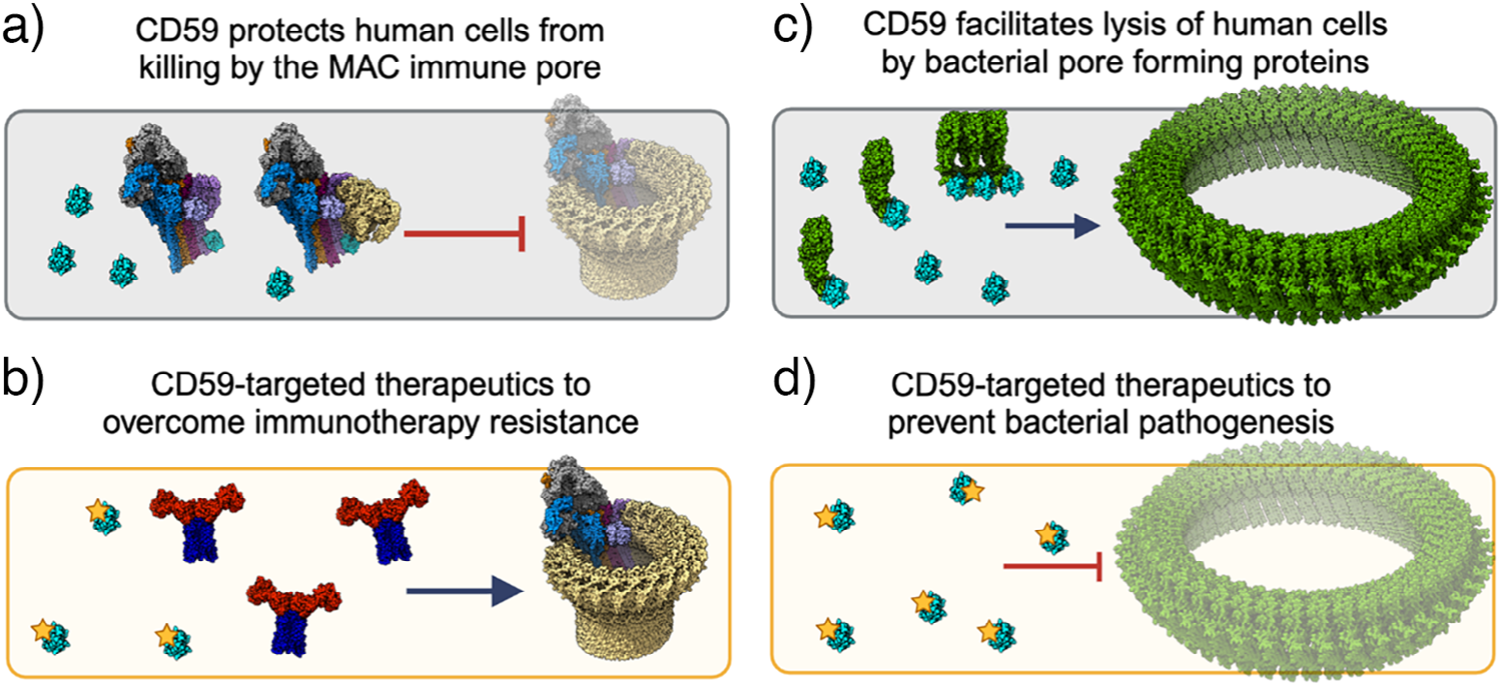
CD59 controls lysis of human cells by regulating pore forming mechanisms. a) CD59 (cyan surface) binds complement components C8α (pink surface) and C9 (tan surface) during assembly of the complement MAC (C5b8 and C5b9) to prevent damage to human cells by MAC (transparent surface). b) Cancer cells evade complement killing by over‐expressing CD59. CD59‐therapeutics (star) are needed to resensitize these cells to immunotherapies such as rituximab, an anti‐CD20 complement activating antibody (red and blue surface). **c**) Bacterial pore‐forming proteins such as ILY (green surface) co‐opt CD59 (cyan surface) to target human cells. CD59‐binding enables oligomerization of the toxin and triggers conformational changes required for pore‐formation. d) CD59‐targeted therapeutics (star) could also serve as potent antimicrobials by interfering with bacterial virulence mechanisms. Diagrams were generated in ChimeraX using structural data: C5b8‐CD59 (PDB: 8B0F); C5b9‐CD59 (PDB: 8B0G); MAC (PDB: 6H04); CD20‐Fab complex (PDB: 6VJA); ILY‐CD59 monomer (PDB: 4BIK); ILY‐CD59 oligomer (6ZD0); and a model for the ILY pore based on the homologue pneumolysin (PDB: 5LY6). The composite figure was created in BioRender.

CD59 is a cell surface protein that plays an important role in mediating human health and disease. It is anchored to the plasma membrane through a glycosylphosphatidylinositol (GPI) anchor^[^ ^1^ ^]^ and is widely distributed in human tissues and circulating cells.^[^ ^2^ ^]^ CD59 is the only membrane‐bound inhibitor of the complement‐mediated membrane attack complex (MAC), and it is the body's last line of defense against MAC‐mediated cell death (Figure 1a). Complement‐mediated cytotoxicity is an effective mechanism for killing cancer cells, in part through induction of MAC activity,^[^ ^3^, ^4^ ^]^ and over‐expression of CD59 in cancer cells contributes to immunotherapy resistance (Figure 1b).^[^ ^5^, ^6^ ^]^ CD59 is also co‐opted by opportunistic bacterial effectors, which use the receptor to target and lyse human cells (Figure 1c).^[^ ^7^ ^]^ Therefore, developing therapeutics that can regulate CD59 activity has the potential to impact both immunotherapy resistance mechanisms and bacterial pathogenesis (Figure 1b,d).

CD59 inhibits complement by preventing membrane perforation and polymerization of the MAC pore. MAC is formed by stepwise sequential assembly of five different complement proteins: C5b, C6, C7, C8, and eighteen copies of C9.^[^ ^8^ ^]^ During pore formation, complement proteins undergo dramatic structural changes that trigger a transition of transmembrane residues from α‐helix to β‐hairpin,^[^ ^9^ ^]^ associating in the membrane to form a giant β‐barrel pore. CD59 directly binds the alpha chain of C8 (C8α)^[^ ^10^ ^]^ as the β‐hairpins cascade toward the membrane and sterically blocks the transmembrane residues of C9 from accessing the membrane,^[^ ^11^ ^]^ inhibiting pore formation.^[^ ^11^, ^12^, ^13^ ^]^ Consequently, C9 is trapped in a conformation which is not competent to further oligomerize, and lysis is averted (Figure 1a).^[^ ^11^, ^14^ ^]^

In contrast to its role inhibiting MAC pores, CD59 may be hijacked by cholesterol‐dependent cytolysins secreted by Gram‐positive bacterial pathogens, which use CD59 as a receptor on human cells to promote oligomerization of toxins at the membrane. Intermedilysin (ILY) is a pore‐forming virulence factor secreted by *Streptococcus intermedius*,^[^ ^15^ ^]^ and an archetypal example of CD59‐responsive cholesterol cytolysins. CD59 binds these cytolysins through a β‐hairpin extension in their membrane binding domain (domain 4, d4).^[^ ^16^, ^17^ ^]^ CD59‐binding of ILYd4 coordinates oligomerization of membrane‐bound monomers and triggers conformational changes in the pore‐forming domain that facilitate formation of a β‐barrel pore (Figure 1c).^[^ ^18^ ^]^

Given its importance in controlling complement and in bacterial pathogenesis, several studies have investigated how CD59 could be targeted to treat human disease. CD59‐inhibiting antibodies were shown to have a costimulatory effect on T cells,^[^ ^19^ ^]^ highlighting the potential for CD59‐targeting therapeutics in modulating immunotherapies. The development of bispecific antibodies further increased complement‐mediated cell killing by combining complement activation by CD20‐targeting antibodies with monoclonal antibodies (mAbs) against CD59.^[^ ^20^ ^]^ Other strategies using recombinant ILY derivatives (rILYd4) have been explored as potential CD59‐targeting therapeutics. rILYd4 was shown to enhance complement‐dependent cytotoxicity (CDC) in rituximab resistant lymphoma cells and a mouse model system.^[^ ^21^ ^]^ Similar strategies showed promise for inhibiting the enveloped virus HIV‐1, which cloaks itself in CD59 during virus budding.^[^ ^22^ ^]^ Whilst none of these strategies have led to a clinically approved drug targeting CD59, the results underscore the potential of CD59 as a therapeutic target.

Here we report the discovery of the first macrocyclic peptide CD59 ligands, with low nanomolar affinity for CD59. We conducted X‐ray structure analysis of a complex with CD59, which facilitated structure‐activity relationship (SAR) studies and allowed us to identify key residues in the peptide sequence that are essential for binding and activity. Structure‐based design of macrocyclic peptide conjugates led to the identification of the first low molecular weight, dual‐function, cell‐active CD59 inhibitors, delivering both nanomolar inhibition of ILY‐mediated cell lysis and potent enhancement of antibody‐targeted CDC in cell models.

## Results and Discussion

### Discovery of the First Macrocyclic Peptide Ligands for CD59

**Figure 2:**
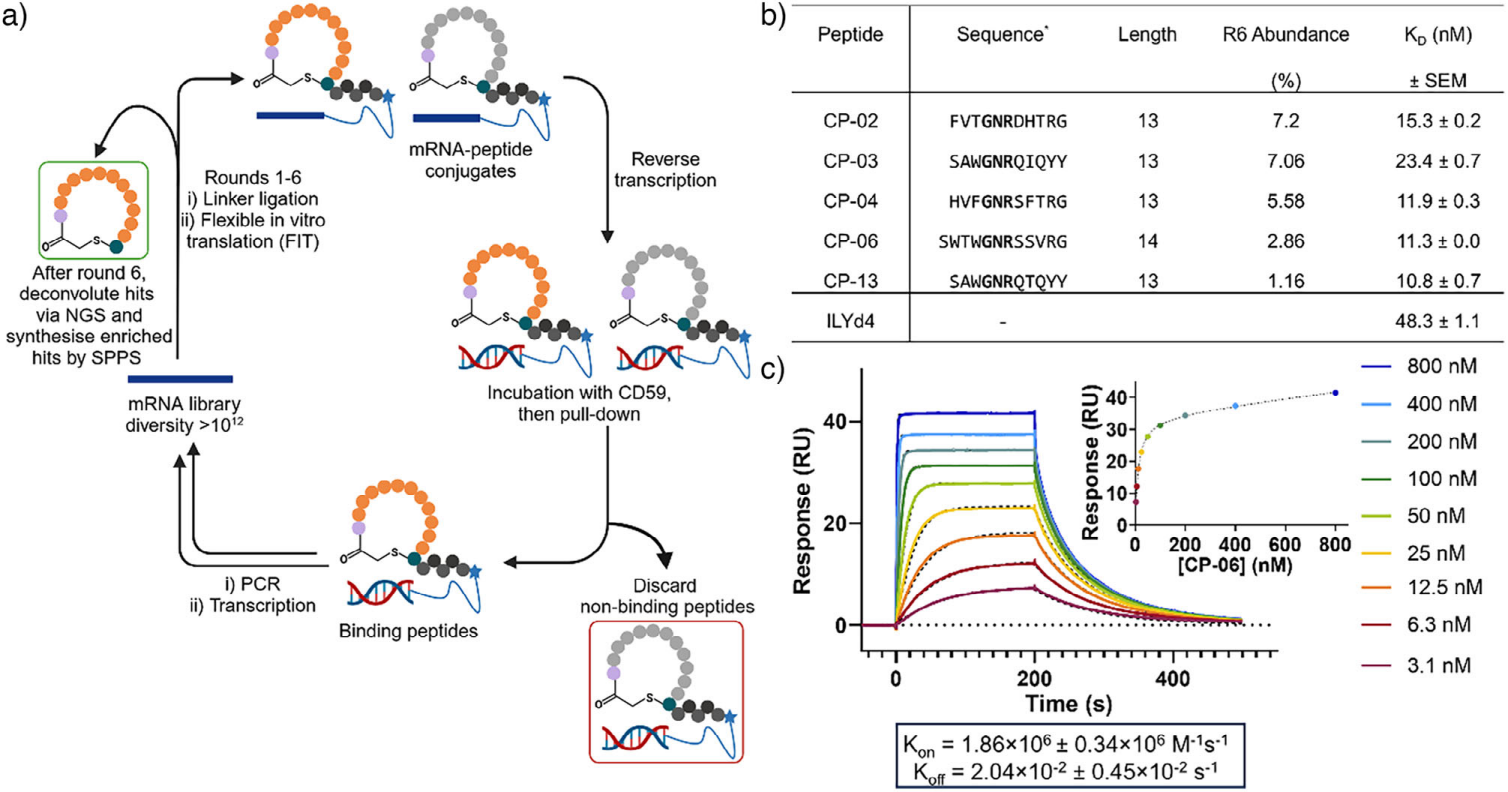
Discovery of macrocyclic peptide ligands with nanomolar CD59 affinity using mRNA display. a) The selection process. PCR = polymerase chain reaction, SPPS = solid phase peptide synthesis. b) Table showing details of the peptides with nanomolar affinity for CD59 as well as the binding affinity of the lead inhibitor of CD59, rILYd4. *K*_D_ values are presented as the average of three technical replicates ± SEM. Structures of all peptides and SPR sensograms are presented in Figures S1 and S2. *indicates the presented sequences are the variable regions of these cyclic peptides; each is flanked by an N‐terminal Ac‐d‐Tyr cyclized to a C‐terminal Cys. R6 abundance (%) is defined as (the given number of NGS reads for a given sequence/the total number of reads of unique sequences observed in NGS)*100 following the 6th selection round. c) Representative sensogram of the interaction between CP‐06 with immobilized CD59 by SPR analysis. Inset showing the equilibrium binding curve of CP‐06 to CD‐59. Below in the box are the association and dissociation rate constants of CP‐06. Concentrations are plotted in descending order from 800‐3.1 nM in line with their position on the legend.

To discover novel non‐protein CD59 ligands, we screened a library of 10^12^ cyclic peptides against CD59 using mRNA‐display (Figure 2a).^[^ ^23^, ^24^, ^25^, ^26^, ^27^, ^28^, ^29^ ^]^ Briefly, an mRNA library was designed in which the start codon was reprogrammed from Met to *N*‐chloroacetyl‐d‐Tyr (ClAc‐d‐Tyr), followed by 10–12 randomly encoded natural amino acids, a Cys residue, a (GlySer)_3_ linker, and a stop codon. Per previous studies discovering macrocyclic inhibitors of protein interactions,^[^ ^24^ ^]^ the starting library comprised a 1:1:1 molar mixture of 12–14 mer macrocycle residues to sample a range of different conformations through varying macrocycle sizes due to previous reports showing macrocycles of these sizes have high affinity and selectivity for their targets.^[^ ^23^, ^24^, ^30^ ^]^ The total library diversity was 1.51 × 10^13^ molecules, comprising 5.02 × 10^12^ molecules of each macrocycle size, covering a subset of the maximum theoretical diversity of this library design. Following in vitro translation, ClAc‐d‐Tyr spontaneously reacts with either the C‐terminal Cys or a Cys in the random region, yielding macrocyclic thioether peptides. Hits from the library were identified through iterative enrichment and amplification against biotinylated CD59, then immobilized on streptavidin beads. Fifteen hit sequences (CP‐01 to CP‐15), enriched with abundance >1% relative to the total pool of recovered binders after the 6th round of selection, were synthesized by solid phase peptide synthesis (SPPS), without the (Gly‐Ser)_3_ linker.

To identify which of these peptides bound CD59 most tightly, we used a Biacore S200 surface plasmon resonance (SPR) platform to investigate CD59 affinity (Figure 2b), using a known protein binding partner of CD59 (rILYd4) as a benchmark. Four 13‐mer macrocyclic peptides and one 14‐mer macrocyclic peptide were identified with a dissociation constant (*K*_D_) lower than that of rILYd4. Considering their fourfold lower molecular weight, these peptides bind with up to 19‐fold greater efficiency compared to rILYd4 (Figure 2b). All five peptides share a Gly‐Asn‐Arg amino acid motif in their sequence and feature an aromatic residue (Phe or Trp) at or near the N‐terminus, suggesting that these common features may be important for CD59 binding (Figure 2b). CP‐06 was selected as the lead hit due to its combination of high affinity (Figure 2c), good solubility, and absence of aggregation, the latter of which was observed with the equipotent hit CP‐13.

**Figure 3:**
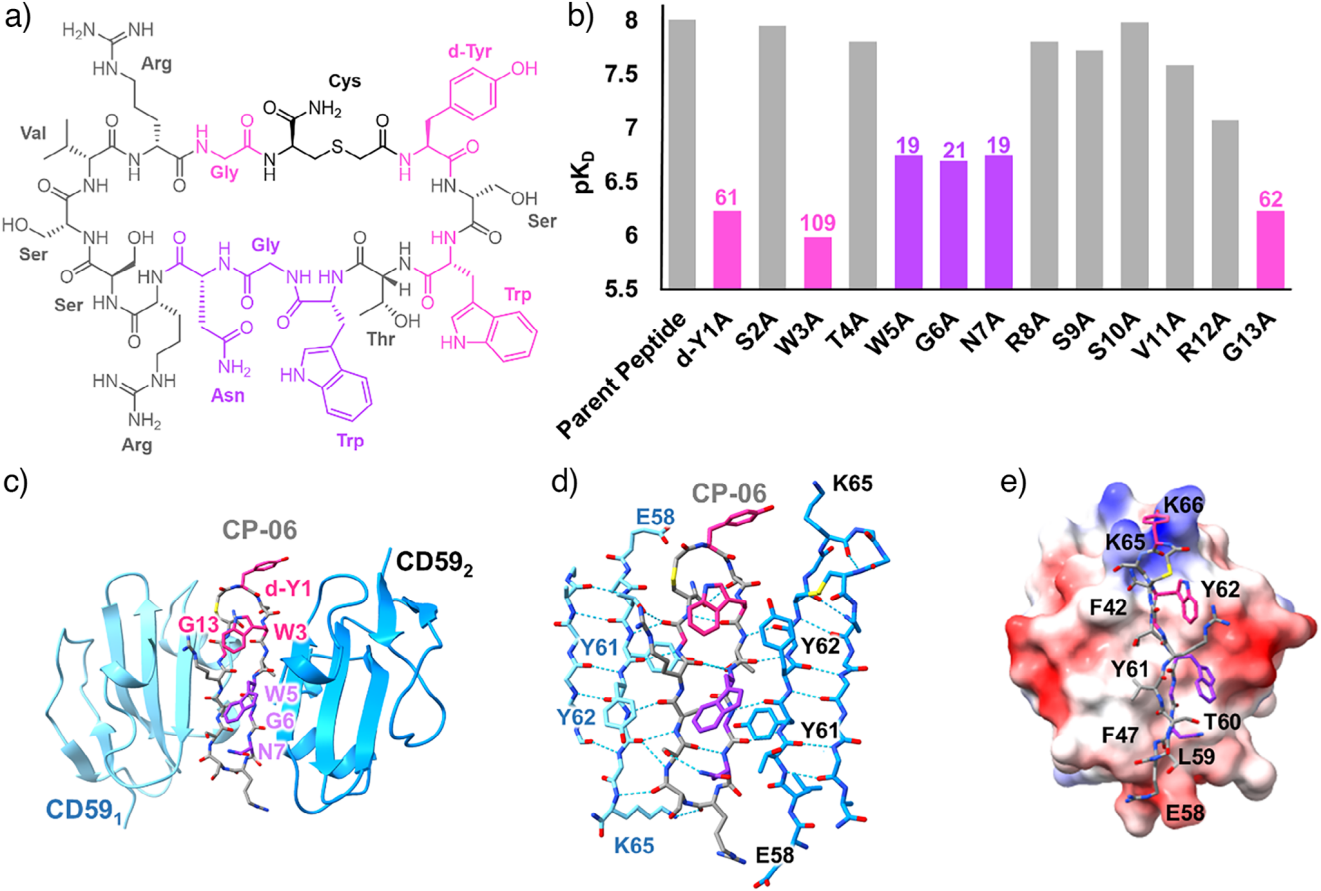
Structure Activity Relationship of CP‐06 and CD59. a) Chemical structure of CP‐06 with the residues colored with a two‐color scale according to impact on *K*_D_ in the Ala scan, grey (minimum) to pink (maximum). b) Histogram of pK_D_ (‐Log_10_(*K*_D_)) values measured by SPR using the same color scheme as in (A). Numbers above columns show fold decrease in *K*_D_, *n* = 1. All sensograms are presented in Figure S2G–S. c) Crystal structure of the CD59:CP‐06 complex (PDB: 8CN6). CP‐06 (sticks representation, colored as in panel *A*) adopts a cyclic β‐hairpin conformation and binds two copies of CD59 (cyan ribbons) through an intermolecular β‐sheet. d) Network of hydrogen bonds (dashed lines) stabilizing the complex. CP‐06 residues are colored according to impact on *K*_D_ in the Ala scan, as in panels *A* and *B*. Sidechains of CD59 interface residues are shown as sticks. e) Coulombic electrostatic potential ranging from−10 (red) to 10 (blue) kcal (mol·e)^−1^ calculated from the CD59 model (surface) of the CD59:CP‐06 complex. CP‐06 is shown as sticks. CD59 contact residues within the binding interface are labeled.

We next undertook a full Ala scan of CP‐06 (Figure 3a) by SPR to further explore the binding interface and identify residues suitable for further modification (Figure 3a,b). We found that substitution of Trp3 for Ala caused a 109‐fold loss in binding affinity, revealing a key role in the interaction. The other two most significant substitutions flanked the cyclization position, d‐Tyr1 and Gly13. Here, a change of either of these residues to Ala resulted in an ∼60‐fold loss in binding affinity. Ala substitutions at Trp5, Gly6, and Asn7 led to smaller but still significant reductions in affinity, while mutations at other residues showed comparable binding affinities to CP‐06 (Figure 3b). A linear analogue of CP‐06 showed a complete loss of binding, confirming the importance of the macrocycle (Figure S2F). Taken together, these data suggest that d‐Tyr1, Trp3, and Gly13 are most important for CD59 binding, with a secondary role for Trp5 and Gly6 and Asn7 from the GNR motif.

### Binding CD59 Through an Intermolecular β‐sheet

To understand the roles of key residues identified by our Ala scan, we used X‐ray crystallography to determine the molecular basis of the CP‐06:CD59 interaction. Crystals of CP‐06 in complex with the ectodomain of CD59 diffracted to 2.4 Å, and the structure was solved by molecular replacement. In the crystal structure, two CD59 molecules sandwich a single macrocyclic peptide (Figure 3c), with three sets of complexes in the asymmetric unit. The macrocyclic peptide forms a β‐hairpin that is stabilized on each side by an extensive network of hydrogen bonds between C‐terminal β‐strands of the adjacent CD59 molecules (Figure 3d). Together they create a large antiparallel intermolecular β‐sheet, in which the two copies of CD59 are oriented in opposite directions.

Residues highlighted by SPR analyses play key structural roles in stabilizing β‐turns of the cyclic peptide β‐hairpin. The unique geometry of the d‐Tyr1 within the cyclic peptide facilitates the β‐turn at the tip of the hairpin (Figure 3d), consistent with a known role for d‐amino acids in structuring short β‐hairpins.^[^ ^31^ ^]^ Furthermore, the cross‐strand pairing of Trp3 with Gly13 (Figure 3d) also likely contributes to peptide secondary structure,^[^ ^32^ ^]^ while Asn7, Arg8 of the GNR motif, and Ser9 form the second β‐turn of the cyclic peptide (Figure 3d). These data, together with the evidence that a linear version of the peptide abolishes binding (Figure S2F), suggest that the β‐hairpin geometry is a major driver for its interaction with CD59.

Despite an extensive H‐bond network with backbone atoms, there are no hydrogen bonds between side‐chain atoms of CD59 and CP‐06. Instead, side‐chain interactions are dominated by hydrophobic contacts involving CD59:F47, T60, L59, Y61, Y62, and F42 (Figure 3d,e). Specifically, the CD59:Y62 side chain intercalates between the peptide Trp3 and Trp5 residues and explains the significant reduction in binding when either peptide residue is substituted for Ala. Other contacts occur at the two β‐turns. CD59:E58 interacts with one end of the β‐hairpin, while both CD59:K65 and CD59:K66 form an interface with the second β‐turn (Figure 3d).

**Figure 4:**
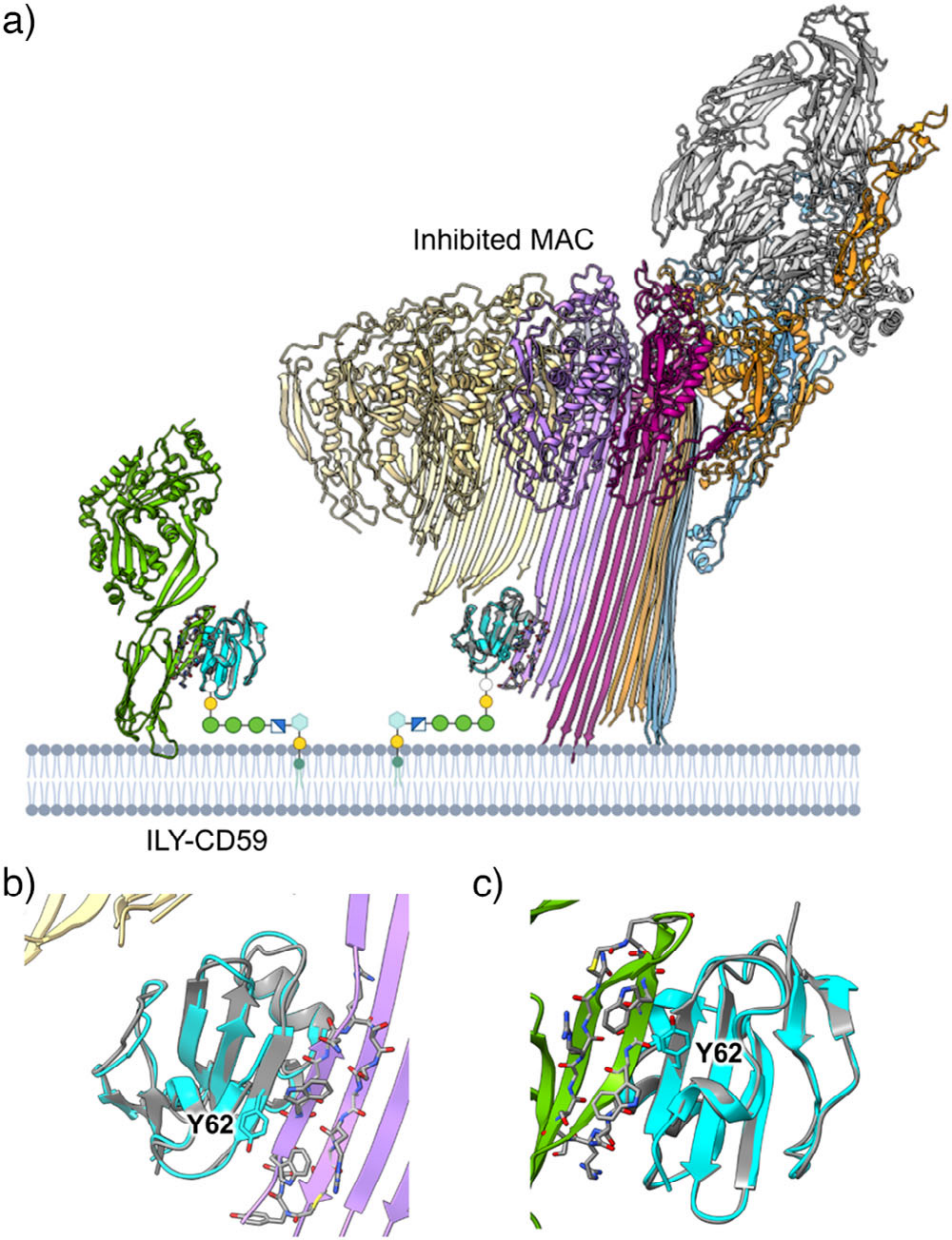
Structural comparisons of CD59‐binding interfaces. a) Structures of CD59–inhibited MAC complex, C5b8‐CD59 (PDB: 8B0F), and ILY‐CD59 complex (PDB: 4BIK). Proteins colored as in Figure 1b,c. b), c) Superposition of CD59:CP‐06 with C5b8‐CD59 b) and ILY‐CD59 c) shows how CP‐06 mimics the binding modes of known CD59‐interaction partners. In all three structures, CD59 engages a β‐hairpin structural motif of its binding partner. CD59:Y62 (cyan sticks) plays a key role in all three interactions.

CD59 residues at the peptide‐binding site are also involved in binding cholesterol‐dependent cytolysins and the pore‐forming machinery of MAC. Despite a lack of sequence homology between CP‐06 and the β‐hairpin binding motifs of C8α and ILYd4, CD59 residues CD59:F47, CD59:Y61, CD59:Y62, and CD59:E58, which dominate the CP‐06 interface, contribute to both MAC (Figure 4a,b) and bacterial toxin (Figure 4a,c) binding modes. In all three structures, CD59 binds a β‐hairpin motif through its C‐terminal β‐strand to create an intermolecular β‐sheet (Figure 4b,c). In our structure, the CP‐06 β‐hairpin bridges two CD59 molecules in opposing orientations to continue the anti‐parallel β‐sheet (Figure 3c,d). Therefore, CP‐06 appears to mimic CD59 binding modes for both immune proteins and bacterial virulence factors.

### Macrocyclic Peptides Inhibit CD59 Function in Cell Models

**Figure 5:**
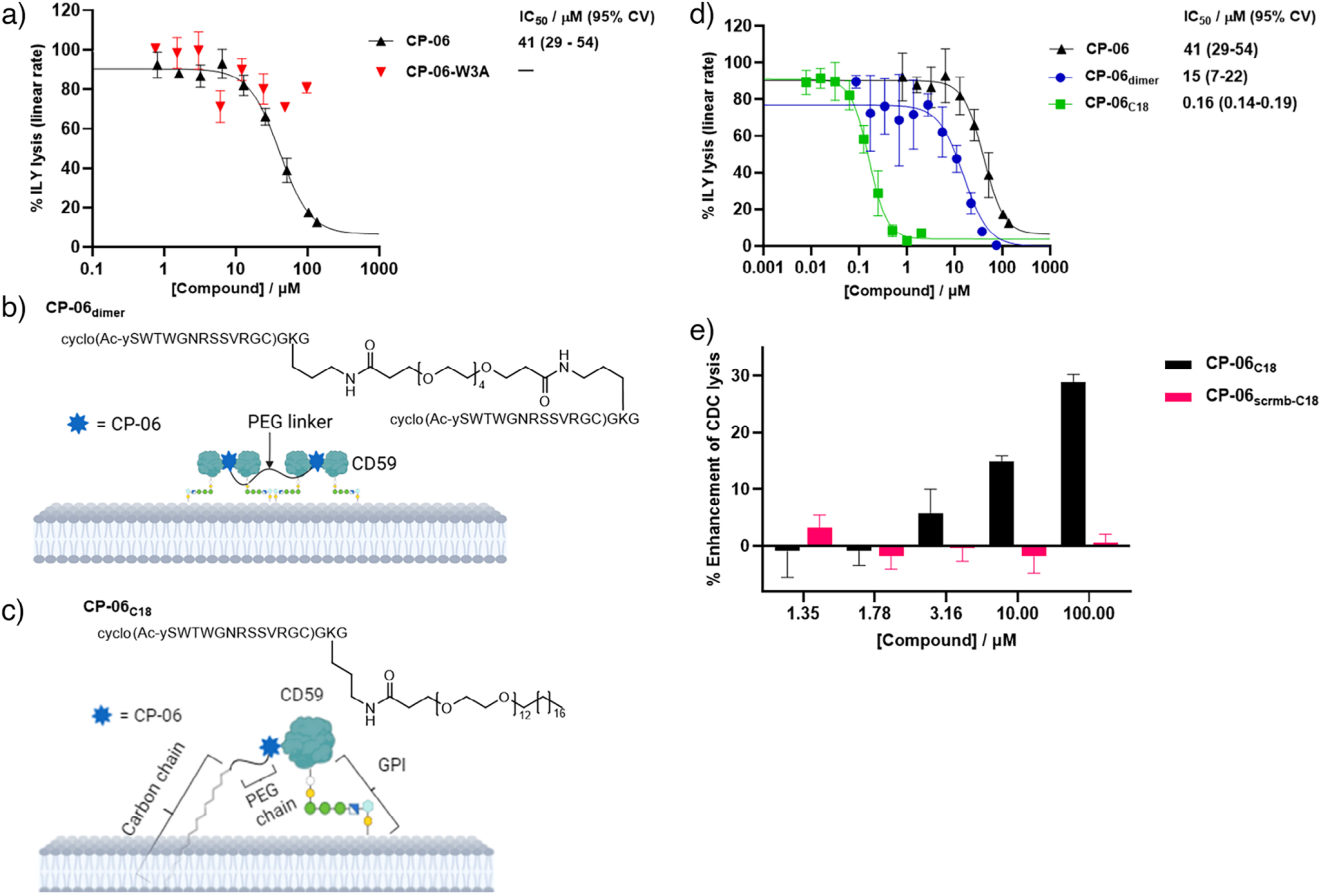
CP‐06 and optimized analogues control CD59 activity in cellular assays. a) ILY lysis assay. SK‐BR‐3 cells were treated with CP‐06 (*n* = 4) or CP‐06[W3A] (*n* = 2), then with Sytox green dye and ILY (625 µM). The plate was imaged immediately with the IncuCyte S3 Live Cell Analysis System and then every hour up to 14 h. The linear phase of lysis was taken from each well, and linear regression was performed to extract individual slope values, which were then normalized to maximum lysis rate (Sytox and ILY) vs. 0% lysis (Sytox only) within each plate. Percentage lysis is plotted as the mean of biological replicates ± SEM against [compound], and the data are fitted to a four‐parameter dose response function, generating an IC_50_ value for each compound. IC_50_ values are reported with 95% confidence intervals in brackets. b), c) Chemical structure and cartoon representation of CP‐06_dimer_ b) and CP‐06_C18_ c). d) IC_50_ values for CP‐06 (black, *n* = 4), CP‐06_dimer_ (blue, *n* = 3), and CP‐06_C18_ (green, *n* = 3) in the ILY lysis assay.  IC_50_ values are reported with 95% confidence intervals in brackets. e) Complement dependent cytotoxicity assay. Human erythrocytes were sensitized with 1% w/v anti‐human erythrocyte Ab with 5% v/v human serum in the presence of a serial dilution of CP‐06_C18_ (black bars) or CP‐06_scrmb‐C18_ (red bars) Percent lysis enhancement was calculated by normalization to 0% enhancement (buffer well) and plotted as the mean of three biological replicates ± SEM.

We next sought to test the ability of CP‐06 to inhibit the two known cellular functions of CD59. As discussed above, the bacterial effector ILY co‐opts CD59 on human cells to form pores.^[^ ^7^ ^]^ We used a human breast cancer line, SK‐BR‐3, which strongly expresses CD59^[^ ^33^ ^]^ to assess lytic activity of ILY and to establish a time course assay to quantify rescue of cells from CD59‐dependent lysis. We found that incubation of these cells with CP‐06 resulted in complete inhibition of ILY lysis with an IC_50_ of 40 µM (Figure 5a). Consistent with our SAR analysis, CP‐06[W3A] showed no inhibition of ILY‐mediated lysis up to the highest tested concentration (100 µM) (Figure 5a). Despite the capacity of CP‐06 to inhibit ILY lysis, there was a substantial drop off in activity compared to its biochemical affinity. We speculated that this may be attributed in part to the complexity and irreversibility of pore formation in the cellular assay. CP‐06 binds to CD59 with relatively rapid reversibility (high on‐ and off‐rate, Figure 2c) whereas CD59‐dependent ILY pore formation is both rapid and effectively irreversible. Therefore, we hypothesized that a higher local concentration of CP‐06 had the potential to enhance inhibition of this process.

Given that CD59 coordinates oligomerization during pore‐formation,^[^ ^18^, ^34^ ^]^ we next explored if dimerization of our CP‐06 peptide might improve its functional activity. During the selection process, we included a C‐terminal linker and encoding mRNA. We therefore hypothesized that modifications external to the macrocycle at the CP‐06 C‐terminus would be tolerated for later peptide functionalization, consistent with the observation that addition of a C‐terminal GSGSGSK tag to CP‐06 showed comparable *K*_D_ (18 nM) to the parent peptide (Figure S2T). Thus, we used a PEG linker at the C‐terminus of CP‐06 to dimerize the peptide (Figure 5b), implementing a strategy known to enhance activity of Pegcetacoplan, a clinically approved peptide therapeutic that targets complement.^[^ ^35^ ^]^ We synthesized CP‐06_dimer_ (Figure 5b) by crosslinking the Lys side chain in a Gly‐Lys‐Gly motif added at the C‐terminus of the monomer via a symmetric PEG_4_ dicarboxylic acid. CP‐06_dimer_ completely inhibits ILY lysis with a 3‐fold enhanced IC_50_ (15 µM), showing a modest synergy over monomeric CP‐06 (Figure 5d). These data confirmed that C‐terminal modification of the peptide does not negatively impact binding and that while dimerization did improve affinity for CD59, other modifications appear to be required to achieve a step change in activity.

Lipidation is a further strategy used to modulate pharmacokinetic properties of peptide ligands, including in the blockbuster GLP1R agonists Liraglutide and Semaglutide.^[^ ^36^ ^]^ We hypothesized that a lipidated CP‐06 analogue, CP‑06_C18_, could enhance local membrane CP‐06 concentration (Figure 5c). We functionalized CP‐06 with a PEG_12_‐C18 group at the Lys residue of the C‐terminal Gly‐Lys‐Gly motif, using a lipid chain similar to that found in GPI anchors, alongside a PEG spacer to balance the hydrophobicity of the lipid. This lipidated peptide showed a dramatic improvement in inhibition of ILY‐mediated cell lysis compared to CP‐06, with an IC_50_ of 160 nM, 250‐fold more potent than CP‐06 (Figure 5d). By contrast, CP‐06_scrmb‐C18_, a scrambled macrocyclic peptide with the same amino acid composition and C‐terminal modification as CP‐06_C18_, showed significantly reduced ILY lysis inhibition (Figure S3), highlighting the importance of both the macrocycle and the lipid for activity.

Having confirmed that CP‐06 competes for a functionally relevant CD59 binding interface, we next explored the ability of CP‐06 to regulate complement activity on cells. In this context, and in diametric contrast to its role in promoting ILY‐mediated pore formation, CD59 inhibits the lytic activity of MAC. As discussed above, MAC‐mediated cell death plays a role in CDC, and inhibition through upregulation of CD59 is a resistance mechanism against immunotherapy in cancer.^[^ ^37^ ^]^ As a proof of concept, we tested the ability of our lipidated peptide (CP‐06_C18_) to enhance cell death of antibody‐sensitized human erythrocytes. This is a tractable cellular model system for CDC inhibited by CD59 on the surface of the erythrocytes, since CD59 activity can be assayed independently from the confounding influence of protein trafficking and endocytosis. We incubated erythrocytes with either CP‐06_C18_ or the scrambled version, CP‐06_scrmb‐C18_, followed by anti‐red blood cell antibody (anti‐RBC) to prime attack by complement, and then added human serum containing complement to initiate CDC through MAC formation. Consistent with inhibition of CD59 in this model, CP‐06_C18_ enhanced mAb‐mediated CDC in a dose‐dependent manner, while no enhancement was observed for CP‐06_scrmb‐C18_. (Figure 5e). Neither peptide caused lysis above background in the absence of anti‐RBC (Figure S4).

## Conclusion

Given the importance of its immunomodulatory role, there is significant therapeutic potential for targeting CD59 in the treatment of cancers and infectious diseases. By focusing primarily on antibody and protein‐based blockade,^[^ ^19^, ^20^, ^22^, ^38^ ^]^ implementation of previous CD59‐targeting strategies has been limited by issues with delivery and immunogenicity. Cyclic peptide drugs are a rapidly emerging new modality acting on extracellular targets and can be administered by injection to treat a wide range of human diseases.^[^ ^39^ ^]^ Cyclic peptides typically show improved pharmacokinetic and pharmacodynamic properties over linear peptides^[^ ^40^ ^]^ and offer an exciting opportunity for novel therapeutics that target CD59. Here, by combining encoded library screening with synthetic chemistry, structural biology, and cellular assays, we validated the first macrocyclic peptide ligands and protein‐protein interaction inhibitors for CD59. Our structural data supported the design of inhibitor conjugates with enhanced activity in cellular assays. The most potent cellular inhibitor, lipidated cyclic peptide CP‐06_C18_, blocks both the MAC pore‐inhibiting and the ILY pore‐augmenting activities of CD59 in cellular assays.

Our data show that secondary structure is crucial for CD59‐binding and that the macrocyclic peptide can be modified to control potency and modulate cellular targeting. We hypothesize that CP‐06 lipidation enhances activity through increasing local concentration at the membrane, consistent with prior reports using similar anchors attached to peptides.^[^ ^41^ ^]^ Future studies could further probe the SARs of these modifications and seek to optimize for stability and selectivity in order to explore more complex physiological models. Furthermore, while our current data shows on‐target binding and inhibition, future work could focus on the application of these ligands to more complex, pathologically relevant systems and off‐target discovery within these systems. Given data showing that C‐terminal and specific residue modifications are well‐tolerated, a biotin handle could be added to the C‐terminus and an appropriate residue upgraded to a photoreactive cross‐linking residue (e.g., photoleucine) for photoaffinity pull‐down/proteomics. We also note that CP‐06 appears amenable to conjugation strategies to facilitate delivery, and future elaboration into novel conjugates might be implemented to tune CD59 activity, such as bifunctional LYTACs to induce CD59 degradation.^[^ ^42^ ^]^ The wide cellular distribution of CD59 combined with recently emerging cellular functions^[^ ^43^, ^44^ ^]^ poses both challenges and opportunities for therapeutic targeting. Our work provides proof of concept for future development of macrocyclic peptides that tune the lytic activity of the complement terminal pathway and potential therapeutics that prevent destruction of host tissues by bacterial virulence factors.

## Author Contributions

E.W.T. and D.B. carried out conceptualization and were responsible for funding acquisition. A.B.P. and S.H. performed data curation. R.M.M., S.H., E.C.C, and K.E.O. conducted formal analysis. R.M.M., J.K.B., A.I.S.A., and A.B.P. carried out the laboratory investigation. T.E.M. carried out the cyclic peptide selection and next‐generation sequencing (NGS) analysis. E.W.T., D.B., and A.K. provided resources. E.W.T., D.B., and A.K. provided supervision. D.B., S.H. performed data visualization. J.K.B., A.I.S.A., E.W.T., and D.B. were responsible for writing the original draft. All authors participated in the writing, review and editing.

## Conflict of Interests

E.W.T. is a founder and shareholder in the companies Exactmer Ltd, Myricx Bio and Siftr Bio, and consultants for or has recently received research funding from Kura Oncology, Dunad Therapeutics, Pfizer, Samsara Therapeutics, Myricx Bio, Merck Sharp and Dohme (MSD), Exscientia and Daiichi Sankyo.

## Supporting information



Supporting Information

Supporting Information

## Data Availability

The data that support the findings of this study are available in the supplementary material of this article.
