# Supplementary material for: Macrocyclic Peptide Probes for Immunomodulatory Protein CD59: Potent Modulators of Bacterial Toxin Activity and Antibody‐Dependent Cytotoxicity
Source: Angew Chem Int Ed Engl. 2025 May 5;64(27):e202422673. doi: 10.1002/anie.202422673 (PMC12207370; doi:10.1002/anie.202422673)
Supplement: Supplementary file 1 — Supporting Information [file ANIE-64-e202422673-s001.docx]

Macrocyclic Peptide Probes for Immunomodulatory Protein CD59: Potent Modulators of Bacterial Toxin Activity and Antibody-Dependent Cytotoxicity

**Authors**

Jasmine K. Bickel^,^ Ammar. I. S. Ahmed, Aidan B. Pidd, Rhodri M. Morgan, Tom E. McAllister, Sam Horrell, Emma C. Couves, Hemavathi Nagaraj, Edward J. Bartlett, Kamel El Omari, Akane Kawamura, Doryen Bubeck, Edward W. Tate

Supporting information.

Table of Contents

1. Supplementary Figures …………………………………………………………………………2

2. Chemistry Methods………………………………………………………………………………7

3. Biological Methods.......………………………………………………………………………..11

4. Appendix: Peptide HPLC traces and Tables S2, 3 and 4……………………………………18

5. References ……………………………………………………………………………………..46

**1. Supplementary Figures**

Figure S1 - structures of enriched hit from the RaPID screen.

Figure S2 - SPR sensograms for enriched hits, CP-06 Ala-scan, CP-06 linear, scrambled sequences and CP-06_C18_. Concentrations are plotted in descending order from the highest to lowest concentration in line with their position on the corresponding legend.


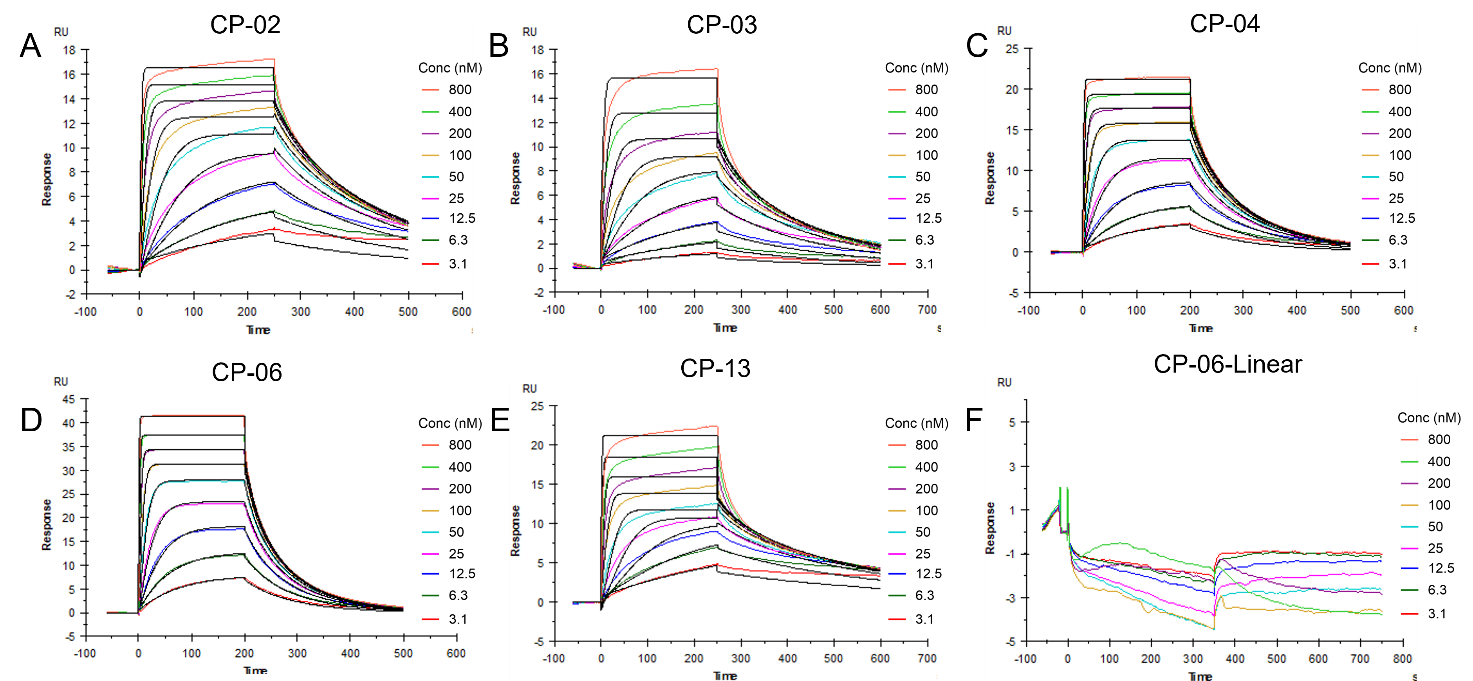


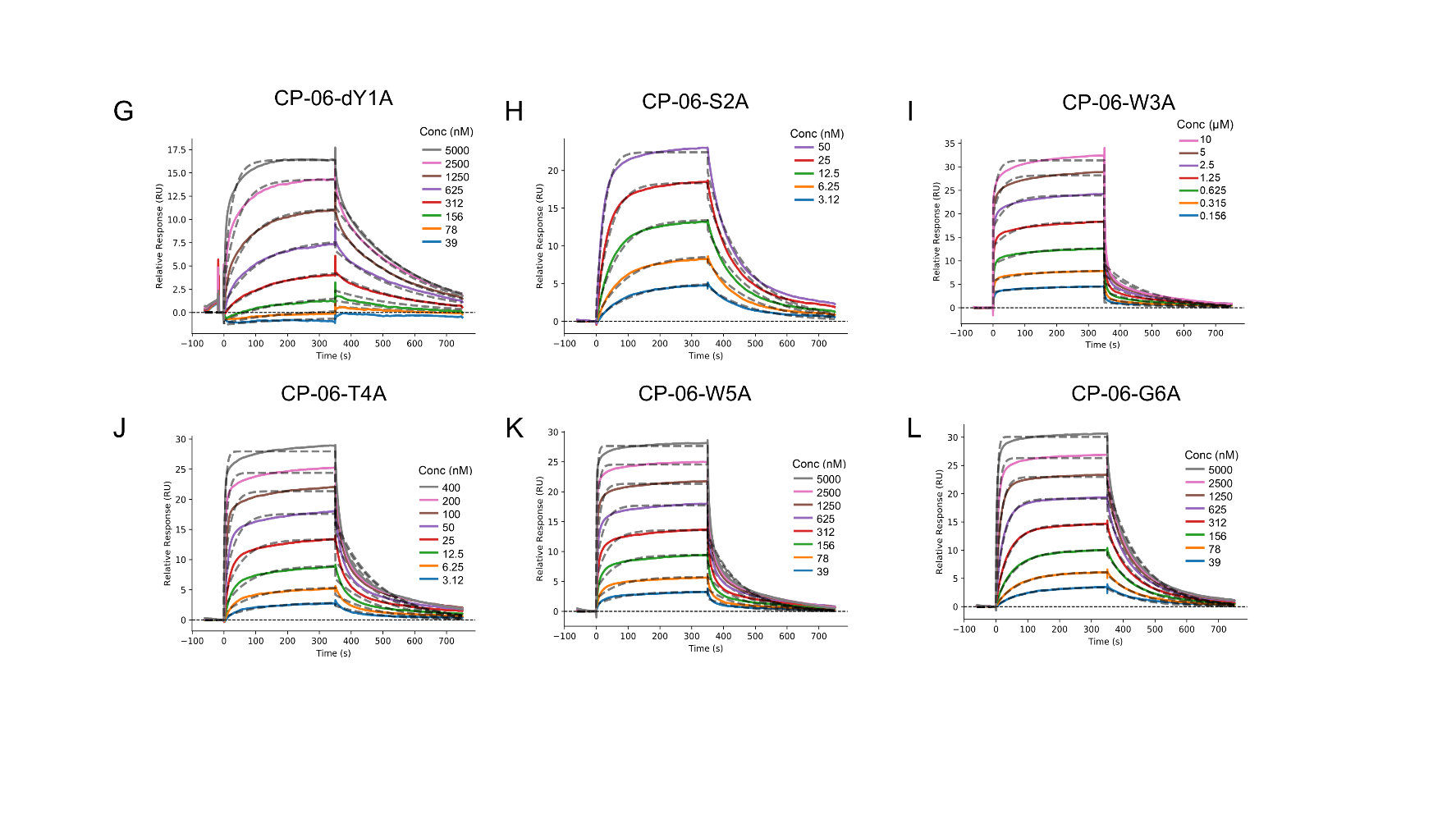


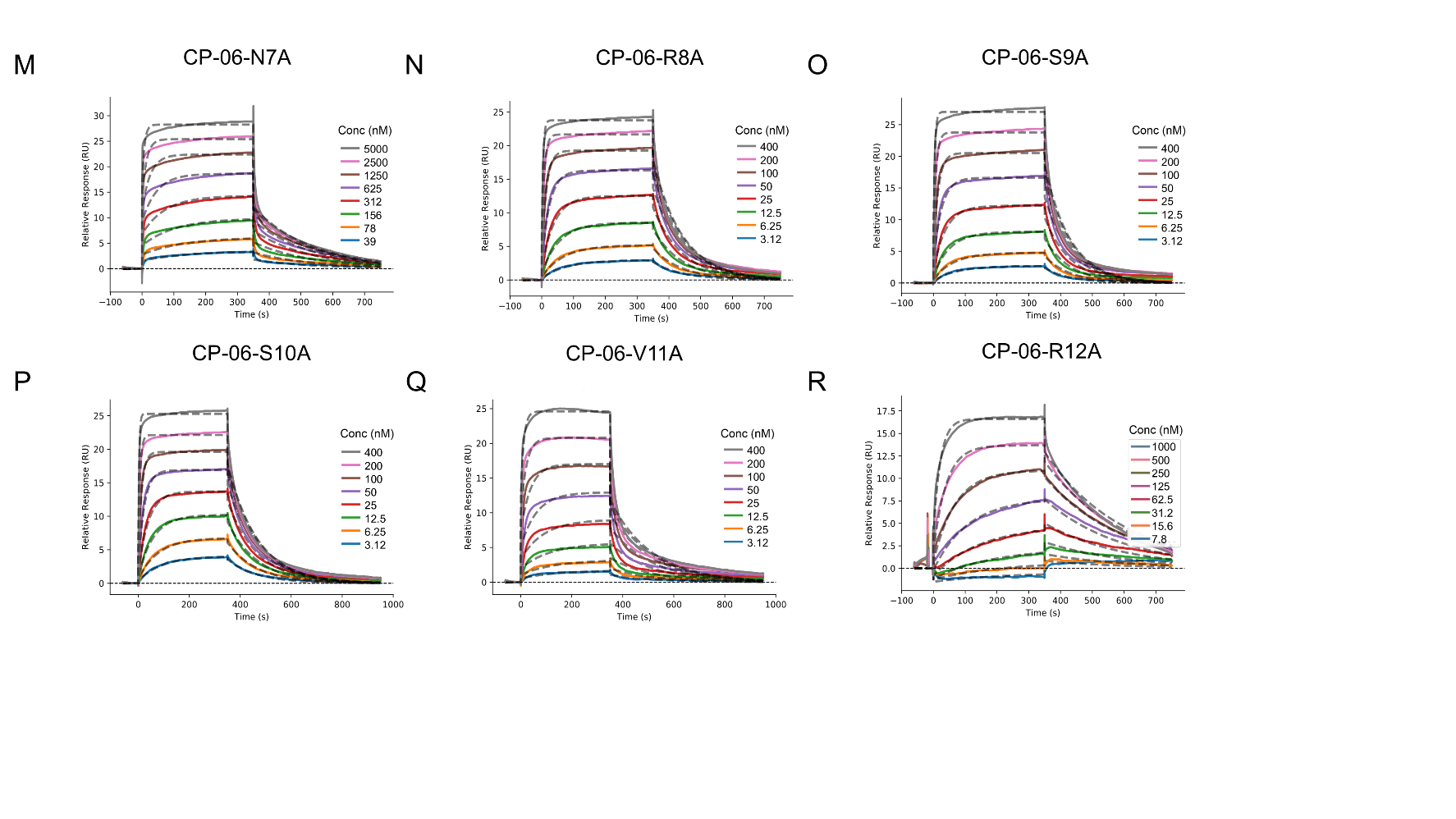


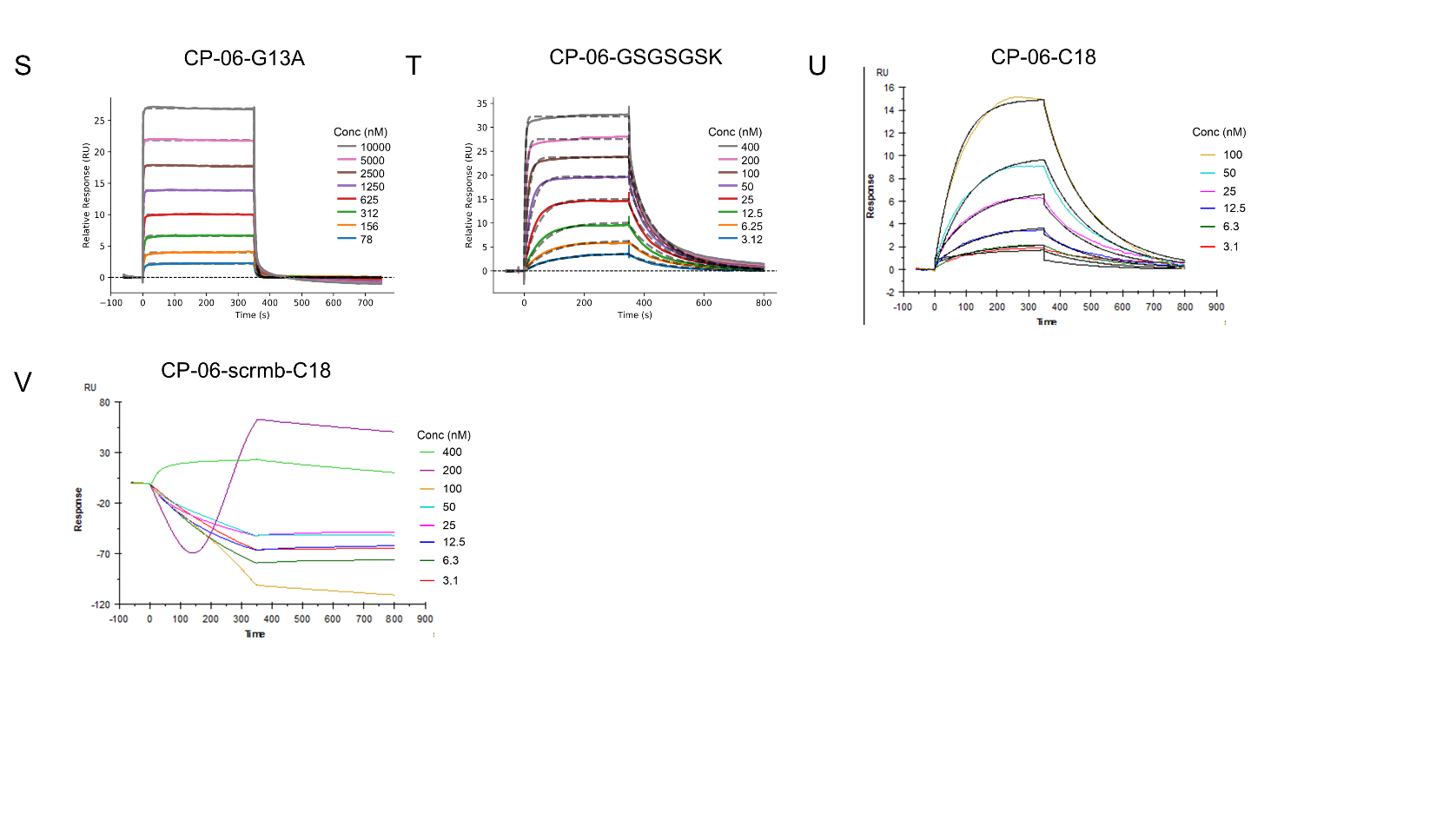


Table S1 – the calculated K_D_ values of CP-06 and it’s Ala-scan analogues

| **Peptide** | PK_D_ (nM) | K_D_ (nM) | Fold Change |
| --- | --- | --- | --- |
| CP-06 | 7.95 | 11 | - |
| CP-06-d-Y1A | 6.17 | 668 | 61 |
| CP-06-S2A | 7.89 | 13 | - |
| CP-06-W3A | 5.92 | 1195 | 109 |
| CP-06-T4A | 7.74 | 18 | - |
| CP-06-W5A | 6.68 | 207 | 19 |
| CP-06-G6A | 6.63 | 235 | 21 |
| CP-06-N7A | 6.68 | 209 | 19 |
| CP-06-R8A | 7.74 | 18 | - |
| CP-06-S9A | 7.66 | 22 | 2 |
| CP-06-S10A | 7.92 | 12 | - |
| CP-06-V11A | 7.52 | 30 | 3 |
| CP-06-R12A | 7.01 | 97 | 9 |
| CP-06-G13A | 6.17 | 678 | 62 |

Figure S3 - CP-06_scrmb-C18_ ILY lysis assay.

*Percentage lysis is plotted as mean of 3 biological replicates ± SEM against [CP‑06_scrmb‑C18_] µM.*

Figure S4 – Lysis enhancement assay ran in the absence of anti-RBC Ab.

*Percent lysis enhancement calculated by normalisation to 0% (buffer only) and plotted as the mean of 3 biological replicates ± SEM.*

**2. Chemistry Methods**

Peptide Synthesis:

Reagents: Fmoc-Lys(ivDde), chloroacetic anhydride, trifluoracetic acid (TFA) peptide grade, triisopropyl silane (TIPS), and Fmoc-d-Tyr(tBu)-OH were from Fluorochem (Hadfield, UK). All other Fmoc-protected amino acids and Oxyma pure were from CEM (Buckingham, UK). TentaGel S RAM resin was obtained from Rapp Polymere (Tübingen, Germany. Maleimidohexanoic acid was obtained from Alfa Aesar (Lancashire, UK). Bis-N-succinimidyl-(pentaethylene glycol) ester Bis (NHS)PEG_5_ was obtained from TCI (Oxford, UK). All other chemicals were from Sigma Aldrich unless otherwise stated. All chemicals were used as supplied without further purification. Ultra-pure water was used for the preparation of HPLC solutions and was obtained from a Millipore Elix Q-Gard purification system.

Apparatus: Linear sequences of the peptides were synthesised using Liberty Blue^TM^ Automated Microwave Peptide Synthesizer connected to HT12 resin loader from CEM. Instruments used for the analysis and purification of peptides: (1) Waters RP-HPLC system that composes of Waters 2767 auto-sampler for sample injection and collection; Waters 515 HPLC pump to transfer the mobile phase; XBridge Peptide BEH C_18_ Columns (130 Å, 5 µm, 4.6 mm × 100 mm for analytical runs and 130 Å, 5 µm, 19 mm × 100 mm for preparative runs) coupled to a Waters 3100 mass spectrometer (with ESI in positive and negative modes) and a Waters 2998 Photodiode Array, with detection between 200- 600 nm. (2) Shimadzu LC-20AR preparative HPLC system that composes of Shimadzu SIL-10AP autosampler for sample injection; Shimadzu FRC-10A fraction collector for sample collection; Two Shimadzu LC-20 AR pumps to transfer the mobile phase; Phenomenex Aeris Peptide XB-C_18_ column (150 mm × 21 mm, 5 μm, 100 Å) coupled to Shimadzu SPD-20A UV/Vis detector with detection between 190-900 nm. 3) Agilent 1260 Infinity II LCMS fitted with a G7115A Diode Array Detector, G7130A Column Oven, G7129A autosampler, G7112B binary pump and G6125B single quadrapole MSD with a Poroshell C_8_ column (InfinityLab Poroshell 120 EC-C_8_, 3.0 × 50 mm, 1.9 µm). 4) Shimadzu LC-2030C 3d plus fitted with an Aeris Peptide 3.6 µm column. Following HPLC purification of the peptides, the pure fractions were concentrated using Genevac EZ-2 Plus Centrifugal Evaporator, the concentrated fractions were then frozen in liquid nitrogen and were lyophilised with an Alpha 2-4 LD Plus freeze-dryer (Christ).

General Synthetic Route: The linear sequences of the peptides were synthesised with an amidated ‘C-terminus’ by Fmoc SPPS on a 50 µmol scale using TentaGel S RAM resin (217  mg, 0.23 mmol/g). Resins were swelled in DMF (10 mL) for 10 min before the start of the synthesis. The cycle of adding one amino acid (aa) to the resins starts with Fmoc deprotection using piperidine (10% in DMF) supplemented with Oxyma pure (0.1 M) to minimise racemisation. This was followed by a washing step using DMF:DCM (4 × 4 mL, 50:50 v/v) then a coupling step using a 5-fold molar excess of the desired Fmoc-amino acid (1.25 mL, 250 μmol, 0.2 M solution in DMF) preactivated with 5 eq. Oxyma Pure (0.5 mL, 250 μmol, 0.5 M solution in DMF) and 10 eq. DIC (1 mL, 500 μmol, 0.5 M solution in DMF). For the addition of arginine, a double coupling was performed, and a double coupling was performed for all the coupling steps after the 10th aa in the sequence regardless of the aa. Standard microwave methods were selected for deprotection and coupling steps for all the aa except for histidine for which a special microwave method was selected for the coupling step. Details of the microwave methods used are summarised below. The process of deprotection/coupling was repeated for each amino acid and automated synthesis ended with a final Fmoc-deprotection step to afford peptides with a free N-terminal amine. At the end of the synthesis, peptides were transferred to a fritted syringe and washed with DMF (3 × 5 mL) then DCM (3 × 5 mL) and stored at -20 °C on resin. Chloro-acetylation of the N-terminus was performed manually as previously described.^1^ Briefly, a solution of chloroacetic anhydride (42 mg, 5 eq., 250 µm) in DMF (2 mL) was added to the resin-bound peptide. The mixture was shaken at RT for 30 min and the completion of the reaction was monitored by LC-MS analysis following a test cleavage of the peptide from resin. The resin was filtered and washed with DMF (3 × 5 mL) and DCM (3 × 5 mL). Next, the peptide was cleaved from the resin with 4 mL of freshly prepared cleavage cocktail (CK) (TFA:TIPS:H_2_O, 95:2.5:2.5 v/v/v). The CK containing the cleaved peptide was collected and a fresh CK (2 × 1 mL) was added to the resin and collected. The combined CK solutions were then concentrated under a stream of nitrogen gas to approximately 1 mL. Next, 10 mL of ice-cold diethyl ether was added to precipitate the peptide. Peptide pellets were collected by centrifugation (4000 rpm, 4 °C, 5 min) and the supernatant ether layer was discarded. Addition of 10 mL cold diethyl ether, centrifugation and discarding of the supernatant was repeated twice more, then the peptide was left in the desiccator overnight to dry. The dried linear peptides were dissolved to 5 mM solution using MeCN:H_2_O mixture (50:50 v/v) and the pH of the solution was adjusted to 8-9 using NaOH (0.1 M) and HCl (0.1 M). The solution was then stirred on a heat block at 60 °C for 40 min to effect cyclisation. The reaction was monitored by LC-MS analysis and after the completion of the reaction, 2-3 drops of formic acid were added to quench the reaction. The peptide solution was then directly purified by RP-HPLC.

Table S2: Microwave settings used during the Fmoc-SPPS of the linear peptides.

| Standard deprotection | | | | |
| --- | --- | --- | --- | --- |
| Stage | Temp (℃) | Power (W) | Time (s) | Delta T |
| 1 | 75 | 155 | 15 | 2 |
| 2 | 90 | 30 | 50 | 1 |
| Standard Coupling | | | | |
| Stage | Temp (℃) | Power (W) | Time (s) | Delta T |
| 1 | 75 | 170 | 15 | 2 |
| 2 | 90 | 30 | 110 | 1 |
| Histidine Coupling | | | | |
| Stage | Temp (℃) | Power (W) | Time (s) | Delta T |
| 1 | 25 | 0 | 120 | 2 |
| 2 | 50 | 35 | 240 | 1 |

Analysis and purification:

The analysis of the peptides was performed on A) Waters LC-MS system, with an injection volume of 20 µL, the flow rate was 1.2 mL/min, and using one of the following solvent gradients: 5 to 98% (AN1) or 20 to 98% (AN2) of eluent B1 (0.1% formic acid (FA) in MeCN) in eluent A1 (0.1% formic acid in H_2_O) over 10 min. All runs were followed by washing the column using 98% of eluent B1 in eluent A1 for 3 min, and finally, re-equilibration of the column to the starting eluent for 5 min. The system was coupled to a Waters 3100 Mass Detector system and data processed using MassLynx 4.1 software B) Agilent LCMS (3) with an injection volume of 1 µL, a flow rate of 0.7 mL/min with a 5-60% gradient of MeCN in 0.1% FA modified H_2_O over 8.5 min. The MSD quadrupole temperature was 100 °C with a gas temperature of 350 °C and a flow rate of 9 l / min and a nebuliser pressure of 35 psig and a skim voltage of 30 V in positive mode. C) Shimadzu LCMS (4) with an injection volume of 15 µL, flow rate of 1.5 mL/min with a 5-95% MeCN (0.08% TFA) in H_2_O (0.08% TFA) over 13 min.

HRMS spectra were recorded on an Agilent 6530 Q-TOF LC/MS ins ESI positive mode using the following parameters: a 1 minute run of 5-95% 5 mM ammonium formate/MeCN through a C18 poroshell 120 column. The source gas temperature was 300 °C at 7 L/min and a sheath gas flow of 12 L/min with a nebuliser pressure of 20 psi., the fragmenter voltage was 100 V. Spectra were analysed using Mass Hunter version 13.09.00.

The purification of peptides was performed either by Waters HPLC system using the following method:

**Prep1**: Injected sample volume between 1 to 5 mL, the flow rate was 20 mL/min, and a solvent gradient from 5 to 98% of eluent B1 in eluent A1 over 22 min. This was followed by a 3 min wash using 98% of eluent B1 in eluent A1, and finally, the column was re-equilibrated to the starting eluent (5% of eluent B1 in eluent A1) for 5 min. A mass-directed fraction collection was used to isolate the desired product.

Or on a Shimadzu HPLC system where injection volumes were 1 to 5 mL, the flow rate was 20 mL/min, detection at 280 nm was used to isolate desired products, and one of the following solvent gradient methods was used:

**Prep2**: 10 to 40% of eluent B2 (0.08% TFA in MeCN) in eluent A1 (0.1% TFA in H2O) over 22 min, 40 to 98% of eluent B1 in eluent A1 over 2 min, 98% of eluent B1 in eluent A1 over 3 min, and finally, the column was re-equilibrated to the starting eluent (5% of eluent B1 in eluent A1) for 5 min.

**Prep3**: 30 to 70% of eluent B2 in eluent A1 over 22 min, 70 to 98% of eluent B1 in eluent A1 over 2 min, 98% of eluent B1 in eluent A1 over 3 min, and finally, the column was re‑equilibrated to the starting eluent (30% of eluent B1 in eluent A1) for 5 min.

Synthesis of GKG: This peptide was used for the synthesis of CP-06_dimer_ and CP-06_C18_. Therefore, a large-scale synthesis was performed; 300 µmol scale (6 × 50 µmol reactions which were combined following automated synthesis). The general synthetic route described above was used for its synthesis.

Synthesis of CP-06_dimer_:

To CP-06-GKG (10.0 mg, 5.2 µmol) in anhydrous DMF (2.0 mL) was added bis(NHS)PEG_5_ (1.0 µL, 1.9 µmol, 1 mg/µL solution in anhydrous DMSO) and DIPEA (5.3 µL, 31 µmol). The resultant mixture was stirred under nitrogen gas at room temperature for 1 h, and the completion of the reaction was confirmed by LC-MS analysis. Next, the volume of DMF was reduced to ca 0.5 mL using a stream of nitrogen gas and then diluted to 5 mL using MeCN:H_2_O (10:90 v/v) supplemented with 0.1% TFA and purified by RP-HPLC.

Synthesis of CP-06_C18_ and CP-06_scrmb-C18_:

To CP-06-GKG/CP-06-scrmb-GKG (10.0 mg, 5.2 µmol in anhydrous DMF (2 mL) was added C_18_-PEG_12_-NHS (7.5 µL, 7.8 µmol, 1 mg/µL solution in anhydrous DMSO) and DIPEA (5.3 µL, 31 µmol). The resultant mixture was stirred under nitrogen gas at room temperature for 1 h, and the completion of the reaction was confirmed by LC-MS analysis. Next, the volume of DMF was reduced to ca 0.5 mL using a stream of nitrogen gas and then diluted to 5 mL using MeCN:H_2_O (10:90 v/v) supplemented with 0.1% TFA and purified by RP-HPLC.

**3. Biological Methods**

The mRNA-display cyclic peptide screen was performed against CD59-biotin by T.E.M: DNA templates for transcription were generated by PCR in a similar manner to that previously described.^2^ RNA oligonucleotides were transcribed from the DNA templates using T7 RNA polymerase and purified by phenol/chloroform extraction followed by ethanol precipitation. Oligonucleotide concentrations were determined spectroscopically by absorbance at 260 nm. In the first selection round a 1:1:1 molar ratio of NNK10/NNK11/NNK12 was used. The procedure for selection rounds 2-6 is described below; round 1 was conducted at twice the scale.

RNA encoding for the peptide library ligated to a puromycin-modified oligonucleotide was transcribed in vitro (final [RNA] = 2.5 µM) using a customised PURE express kit (lacking release factor 1 and methionine, supplemented with 500 µM initiator tRNA acylated with N-chloroacetyl-d-tyrosine prepared as previously described)^3^ in a final volume of 5.6 µL for 1 hour at 37 °C. The sample was then diluted with 5 × M-MLV buffer (250 mM Tris-HCl pH 8.3 at 25 °C, 375 mM KCl, 15 mM MgCl2, 50 mM DTT; 2 µL), heated at 60 °C for 15 minutes and cooled on ice while the remaining components for the reverse transcription reaction were added to give a final volume of 10 µL and a composition of 50 mM Tris-HCl pH 8.3 at 25 °C, 75 mM KCl, 3 mM MgCl2, 10 mM DTT, 2 µM P2, 500 µM dNTPs, M-MLV RTase 5 U/µL.

This mixture was incubated at 42 °C for 1 hour before being diluted to 500 µL with PBS (10 mM Na-PO4 pH 7.4, 2.7 mM KCl, 137 mM NaCl) and 0.5 µL taken for subsequent use in qPCR. The diluted reaction mixture was concentrated using a 30 kDa cut-off centrifugal filter to < 50 µL, before being diluted up to 500 µL with PBS once more. This concentration process was repeated twice more, the final volume of the retained solution measured and diluted accordingly to give a final volume of 30 µL and a final buffer composition of PBS with 0.05% v/v Tween-20 (PBS-T).

All subsequent manipulations were carried out at 4 °C using pre-chilled buffers. A further 0.5 µL sample was taken for subsequent DNA quantitation by qPCR. The remainder of the solution was used to resuspend 60 µg of streptavidin coated magnetic beads (previously washed in PBS-T) and the resultant slurry gently agitated for 30 minutes before the beads were pelleted using a magnet. The supernatant was transferred to another portion of beads and the process repeated a further 2 times. The beads from each step were ‘washed’ (resuspended in 30 µL of PBS-T, transferred to a fresh tube, pelleted and the supernatant removed) 3 times before being resuspended in 100 µL of PCR buffer (1× NH4 buffer, 2.5 mM MgCl2) and stored on ice.

After the 3rd bead incubation, the supernatant was transferred to a clean tube and the volume measured by aspirating with a pipette. Biotinylated CD59 and PBS-T were added to give a final volume of 30.5 µL and 400 nM final biotinylated CD59 concentration. A 0.5 µL sample was taken for subsequent DNA quantitation by qPCR and the remaining mixture incubated for 1 hour before being used to resuspend 60 µg of streptavidin coated magnetic beads (previously washed in PBS-T) in a fresh tube and the resulting slurry gently agitated. After 5 minutes the beads were pelleted using a magnet, the supernatant removed and the beads washed as described above, finally resuspending in PCR mix (PCR buffer supplemented with 250 µM dNTPs, 500 nM P1 and 500 nM P2).

All bead-containing samples were heated at 95 °C for 5 minutes before pelleting the beads with a magnet and recovering the supernatant to a fresh tube. The amount of DNA in each sample was quantified by qPCR using appropriate standards and the percentage recovery determined by comparison with the samples taken throughout the selection process. DNA recovered from the CD59-containing sample was amplified by PCR using an appropriate number of cycles based on the qPCR result. The DNA was purified by phenol/chloroform extraction and ethanol precipitation and used as the input for the next selection round.

Sequencing of the enriched DNA and subsequent processing was carried as previously described. ^1^

Primers

P1: TAATACGACTCACTATAGGGTTAACTTTAAGAAGGAGATATACATATG

P2: TTTCCGCCCCCCGTCCTAGCTGCCGCTGCCGCTGCCGCA

Expression and purification of CD59: Recombinant soluble human CD59 with an additional C-terminal cysteine residue was expressed in *E.coli*, refolded and purified from soluble inclusion bodies as previously described^4^ and was a generous gift from Richard Smith (Kings College London). CD59 was further purified by size exclusion chromatography using a Superdex-75 column run in 150 mM NaCl, 20 mM Tris-HCl pH 7.5. Aliquots were then flash frozen and stored at – 80 °C until use.

CD59-biotin production: To liberate the C-terminal cysteine for protein modification, purified CD59-Cys (322 µM, 1.0 eq.) in PBS was incubated with tris(2-carboxyethyl)phosphine (TCEP) (644 µM, 2.0 eq.) for 16 h at RT with gentle agitation. The mixture was then washed with PBS (3 × 200 µL) in a centrifugal spin filter (3 kDa MWCO), and subsequently diluted to twice the original sample volume. Maleimide-PEG2-biotin (6.4 mM, 20 eq., CAS: 305372-39-8) was added and the mixture was incubated for 4 h at RT. Upon completion, CD59-biotin was purified by SEC using a superdex 75 (s75) 10/300 column pre‑equilibrated with PBS. CD59-biotin containing fractions were then pooled, concentrated and flash-frozen in liquid nitrogen for storage at -80 °C.

rILYd4 production

Transformation: pTrcHisA bacterial expression vectors containing the gene for rILYd4 with an N-terminal His6-tag were generously gifted by Rodney K. Tweten. The vector (1 µL, 100 ng/µL) was incubated with BL21(DE3) competent *E. coli* cells (50 µL) on ice for 30 min. Transformation was achieved by heat-shocking for 45 sec at 42 °C followed by recovery on ice for 2 min. SOC media (200 µL) was added to the cells and the sample was incubated at 37 °C for 1 h with shaking (200 rpm). The transformation mix was then plated onto a pre‑warmed LB/ampicillin (100 µg/mL) agar plate and incubated overnight at 37 °C. If desired, plasmid recovery and purification was performed using a QIAprep Spin Miniprep Kit as per the manufacturer’s instructions.

LB (6 mL) supplemented with ampicillin (100 µg/mL) was inoculated with a single colony previously transformed with the desired vector and incubated overnight at 37 °C with shaking (200 rpm). The culture (1 mL) was added to LB/ampicillin media (1 L) and incubated at 37 °C with shaking (200 rpm) until an OD_600_ of between 0.6-0.8 was reached (typically 5-6 h). Protein expression was induced by the addition of IPTG (Cf = 0.5 mM) and the cultures were incubated overnight at 18 °C with shaking (180 rpm). Cell cultures were pelleted (3900 rpm, 20 min, 4 °C) and lysed by sonication in ILY buffer (150 mM NaCl, 20 mM Tris-HCl, pH 7.5) containing cOmplete Protease Inhibitors and DNAse I. The lysate was clarified by centrifugation (15,000 rpm, 45 min, 4 °C) and the supernatant was subsequently incubated with cobalt-chelated TALON affinity resin (4 mL) pre-equilibrated with ILY buffer for 2 h at 4 °C. The resin was washed with ILY buffer (10 CV) followed by ILY buffer containing 10 mM imidazole (5 CV). The immobilised His-tagged protein was then eluted via an imidazole gradient (100-500 mM) prepared in ILY buffer (6 CV combined). Further purification was achieved by SEC on a s75 10/300 column. Purity was assessed by SDS-PAGE analysis and the desired protein-containing fractions were pooled, concentrated using appropriate centrifugal filter units, and flash-frozen in liquid nitrogen for storage at -80 °C until use.

His-Tag cleavage

The imidazole was removed by a HiTrapTM desalting column (5 mL) pre-equilibrated with ILY buffer. The protein-containing fractions were combined and incubated with fresh affinity resin (4 mL) pre-equilibrated with ILY buffer for 2 h at 4 °C with agitation. The flow-through was collected and the resin was re-suspended in a solution of enterokinase (EK) (0.5 U/µL) in EK buffer (150 mM NaCl, 20 mM Tris-HCl, 2 mM CaCl2, pH 7.5) and incubated overnight at RT with agitation. The resin was washed with ILY buffer containing 10 mM imidazole (2 x 2 CV) to collect the His-tag cleaved rILYd4. Any remaining His-tagged protein was collected by elution from the column with ILY buffer containing 100 mM and 500 mM imidazole (4 CV and 2 CV, respectively) and subsequently flash-frozen in liquid nitrogen for storage. The progress of the cleavage reaction and affinity resin purification were assessed by SDS-PAGE analysis. His-tag cleaved rILYd4 was purified further by SEC on a s75 10/300 column pre-equilibrated with PBS and the purity was analysed by SDS-PAGE. Pure protein fractions were pooled, concentrated and flash-frozen in liquid nitrogen for storage at -80 °C until use.

rILYd4 analysis


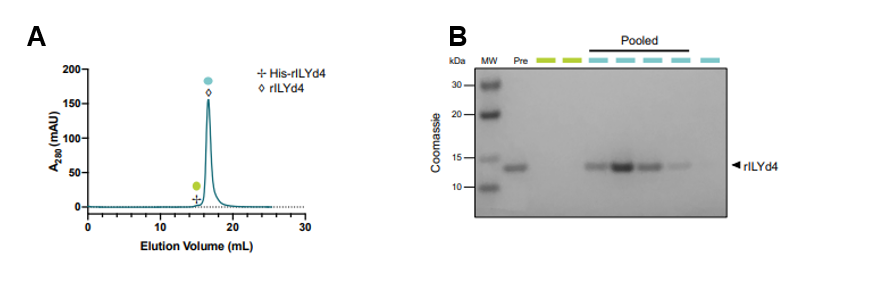


Figure S5 – analysis of rILYd4. (**A**) SEC profile for ‘polish’ purification of pooled fractions performed on a s75 10/300 column pre-equilibrated with PBS buffer at 4 °C. Coloured dots denote peaks analysed by SDS-PAGE. (**B**) SDS-PAGE analysis of peak fractions from SEC ‘polish’ purification. Prepared with a 10% acrylamide gel under non-reducing conditions and performed in MOPS running buffer. Coloured bands correspond to SEC peaks. Pooled fractions containing purified rILYd4 are indicated.

SPR experiments

Equipment: SPR analysis was performed on a Cytiva Biacore S200. All buffers were prepared using ultra-pure water from a Millipore Elix Q-Gard purification system. Solutions and buffers were filtered with a 0.22 μm filter and degassed prior to use. The assays were performed on Series S streptavidin (SA) sensor chip (Cytiva). PBS-P+ buffer 10X (0.2 M phosphate buffer with 27 mM KCl, 1.37 M NaCl and 0.5% Surfactant P20 (Tween 20)) was used for the preparation of the assay running buffer (PBS-P+ 1X).

Immobilisation of CD59-biotin: Biotinylated CD59 was immobilised on the surface of a SA chip by non-covalent capture (biotin streptavidin interaction) as per the manufacturer’s instructions. The system was initially primed with running buffer and normalised using BIAnormalizing solution (70% (w/w) glycerol) (Cytiva). Before immobilising the protein, the surface was conditioned with three consecutive one-minute injections of 1 M NaCl in 50 mM NaOH. Biotinylated CD59 (0.5 µg/mL in running buffer) was flowed over the protein (active) channel for different contact times to achieve different immobilisation levels (600–1200 RU). After each protein injection, the needle and injection loop were washed using 50% isopropanol in 1 M NaCl and 50 mM NaOH. A reference channel was prepared by similar treatment; however, running buffer alone rather than CD59-biotin in running buffer was flowed over the chip the same way.

Interaction analysis: Multicycle kinetic and affinity approach was used for the analysis of peptides interactions with CD59. Two-fold serial dilutions of each peptide were prepared in running buffer and were flowed over both the ligand and reference channel using a flow rate (30 μL/min) at 25 °C. A zero concentration was added to each analyte series and three cycles of buffer were run between analytes to restore the baseline. The obtained sensorgrams were analysed using the embedded Biacore S200 Evaluation software. The data were normalised by subtracting RU values of the reference channel and zero concentration of the analyte series. Kinetic rate constants (k_on_ and k_off_) were determined by fitting the normalised data using the built-in Kinetic analysis model (non-linear regression with a 1:1 Langmuir binding model), K_D_ values were then calculated from the two rate constants (K_D_ = k_on_ and k_off_). The K_D_ values were determined again by fitting the normalised data using the built-in Affinity analysis model (1:1 Langmuir binding model).

Structural Methods

Preparation of the CD59s/CP-06 Complex: An aliquot of CD59 (1.5 mL, 3.0 mg/mL) was purified by SEC using a s75 10/300 column pre equilibrated in crystallisation buffer (150 mM NaCl, 20 mM Tris-HCl pH 7.5). CD59 containing fractions were pooled and concentrated to ca. 2.5 mg/mL using an Amicon Ultra centrifugal filter (3 kDa MWCO). A solution of peptide CP-06 in crystallisation buffer (450 µM) was added to CD59 in 1.2 eq. molar excess and the mixture was incubated for 30 min at room temperature. The complex was then concentrated to ca. 10 mg/mL CD59.

Crystallization and data collection: Crystallisation trays were prepared using a Mosquito (TTP Labtech) liquid handling platform whereby Equal volumes of preformed CP-06:CD59 complex (10mg/ml in 150 mM NaCl, 20mM Tris [pH 7.5]) and precipitant (0.01 M ZnCl_2_, 0.1 M HEPES pH 7.0, 20% w/v PEG 6000) were mixed. Crystals were grown by vapor diffusion at 20 °C in 96-well plates with 0.2 µl droplets. Crystals were cryoprotected with the reservoir solution supplemented with 30% ethylene glycol before cryo-cooling in liquid nitrogen Diffraction data were collected on beamline I04 at Diamond Light Source, Didcot, Oxfordshire, UK, using an Eiger2 XE 16M detector under cryogenic conditions (Diamond BAG proposal number mx17221).

Structure determination and refinement: Diffraction data were processed using Xia2.Dials^5^ and Aimless^6^ to a high-resolution limit of 2.43 Å based on a CC1/2^6,7^ of 0.3 in the highest resolution shell. Detailed data reduction statistics are presented in Table S2. The crystal structure was phased by molecular replacement using Phaser^8^ and the PDB 2UWR^4^ as a starting model. The structure was refined with REFMAC5^9^ using automatic TLS parameters and NCS restraints. Manual refinement and model building performed using COOT 0.9^10^. Model validation was performed using Molprobity^11^ with a final R_factor_/R_free_ value of 23.5%/29.9% and displayed good geometry. Detailed refinement statistics are presented in Table S2. The model and structure factors have been deposited in the RCSB PDB with accession code 8CN6. Structural figures were prepared using ChimeraX.^12^

Cell Biology

Reagents and apparatus: Anti-human RBCs Ab was purchased from Cambridge Bioscience Ltd. Complement serum was purchased from Sigma Aldrich (S1764-1 mL) and resuspended and stored as per manufacturer instructions. The absorbance of the released haemoglobin was measured using Envision Xcite plate reader (PerkinElmer). ILY lysis assay in SK-BR-3 cells was performed using IncuCyte^®^ S3 Live Cell Analysis System (Sartorius).

Tissue culture: All cell culturing was carried out in Class 2 Biosafety Cabinet under strict aseptic conditions. Cells were grown at 37 °C in a 5% CO_2_ incubator. Passage number was kept below twenty. Routine Mycoplasma testing was performed (at least once a month).

SK-BR-3 cells: These were purchased from ATCC and were grown in high glucose Dulbecco’s Modified Medium (41966029, ThermoFisher) supplemented with 10% (v/v) heat-inactivated fetal bovine serum (FBS; Gibco, 10270). Cell harvesting for maintaining the culture and/or seeding for an experiment was achieved by washing the attached cells with PBS followed by the addition of trypsin (0.25% (w/v), 0.5-2 mL depending on the flask size). The cells were incubated at 37 °C, to activate trypsin, until all cells are detached (~ 5 min). Growth medium (10-fold trypsin volume) was added to inactivate trypsin. The appropriate volume from this cell suspension was added to new culture vessels. Stocks of early passages (10^6^ viable cells) were cryopreserved in 1 mL FBS supplemented with 10% (v/v) DMSO.

Erythrocytes: These are primary cells that were obtained from human whole blood and were not handled under aseptic conditions. Screened blood from healthy volunteers was purchased from Cambridge Bioscience Ltd. Erythrocytes were separated by centrifugation (2500 rpm, 15 min, 4 °C), the supernatant was discarded, and the erythrocytes were resuspended gently in PBS and centrifuged as before. The wash step was repeated until the supernatant was colourless. The erythrocytes cell volume (100% stock) was resuspended in PBS to obtain a 50% (v/v) erythrocyte stock suspension. The suspension was stored at 4 °C and was used within one week.

Lysis assays

Determination of optimal ILY concentration: 20,000 SK-BR-3 cells were seeded in a 96-well plate (Grenier, 655180) 24 h before treatment. To minimise the effect of media evaporation the final volume of the assay was set at 200 μL, and the outer wells were filled with 200 μL PBS. Cells were washed with PBS then 180 μL of Sytox Green (SG) (Cf = 250 nM 0.1% (v/v) DMSO in serum-free medium) was added. A two-fold serial dilution of ILY was prepared in serum-free medium (Cf = 10 – 2500 pM). 20 μL from each ILY dilution was added to test wells. The following controls were included: (1) a positive control well that contained cells and Tween-20 (Cf = 1%), (2) a stained untreated control which contained cells and SG, and (3) an unstained untreated control which contained cells only. Any bubbles were removed by a de-bubbler and the plate was immediately transferred to IncuCyte® S3 Live Cell Analysis System where each well was imaged 4 times at 10 × magnification with phase contrast and green fluorescence (λex 440–480 nm, λem = 504–544 nm) every hour for 24 h.

The acquired images were analysed using Incucyte 2019B Rev 2® software. To train the software, the following representative images were selected for each plate:

(1) Untreated unstained control at time zero

(2) Untreated stained control at time zero (SG only)

(3) ILY treated stained control at time zero (ILY and SG)

(4) ILY treated stained control at 48 h (ILY and SG)

Parameters were then set as follows: (1) For phase contrast, segmentation was adjusted to 0.9 and the minimum area was set to 100 μm^2^. The rest of the parameters were not changed (2) For Green fluorescence, a Top-Hat method was used for the segmentation and the radius was set at 13 μm. The rest of the parameters were not changed. For some of the assays, the parameters were refined. Data were extracted as a text file from Incucyte 2019B Rev 2® software and analysed using Microsoft Excel and Prism 9. For each well, a Gp value was calculated which is equal to the integrated green intensity per image divided by the phase area per image. These Gp values were plotted as a measure of ILY lysis against time and the points of the linear phase of the plot (usually up to 8 h) were fitted to a straight line. To generate a dose-response curve, the slopes of the lines were plotted against the corresponding concentration. The data were then fitted using sigmoidal 4-PL model. To compare between biological replicates, the slopes were normalised to a maximum of 100% (ILY and SG treated well) and a minimum of 0% (SG only treated well) within the same plate. The normalised data were plotted as the mean of two biological replicates plus/minus the standard error of the mean (SEM). Based on this data, ILY concentration (600 pM) corresponding to EC_50_ was selected for future assays. This lies within the linear phase of the sigmoidal dose-response curve and gives an assay window of 50%.

Assessing peptides’ ability to inhibit ILY lysis: The cells were seeded as described above. Two-fold serial dilutions of test compounds were prepared as a 10 × solution in the desired range. 20 μL from each dilution was added to each test well, this was followed by the addition of 160 μL of Sytox Green (SG) (final concentration (C_f_) = 250 nM 0.1% (v/v) DMSO) and the plate was incubated for 30 min. 20 μL from ILY (C_f_ = 600 pM) was added to test wells. The following controls were included: (1) a positive control well which contained ILY (C_f_ = 600 pM) and SG, (2) a stained untreated control which contained SG, and (3) an unstained untreated negative control well which contained cells only. Any bubbles were removed by a de-bubbler and the plate was immediately transferred to IncuCyte® S3 and images were acquired and analysed as above.

CDC lysis assay: A 50% (v/v) erythrocyte stock suspension was prepared as described above. This was diluted to 2.5% (v/v) working stock. 160 μL from the erythrocyte working stocks was added to each well of a V-bottom 96-well plate (Greiner, 651201). A two-fold serial dilution of Anti-RBCs antibody (C_f_ = 2.5-320 μg/mL) was prepared as 10X solution and 20 μL from each dilution was added to each test well. The antibody was incubated at RT for 20 mins then 20 μL from complement serum (CS) (C_f_ = 5%) was added to test wells. The following control wells were included: (1) a positive control well which contained Tween‑20 (C_f_ = 1%), (2) a negative control well which contained PBS, (3) Anti‑RBCs (C_f_ = 320 μg/mL) to assess the toxicity of the antibody, (4) CS (5%) to assess the toxicity of the CS, (5) a negative control well which contained Anti-RBCs (C_f_ = 320 μg/mL) and Heat‑Inactivated complement serum (HI-CS), and (6) a vehicle only well which contained PBS without erythrocytes to subtract the absorbance of the vehicle. HI-CS was prepared by heating CS at 56 °C for 30 min. The plate was then incubated at 37 °C for 1 h and then centrifuged (2500 rpm, 5 min) to pellet any un-lysed erythrocyte. 100 μL from the supernatant was transferred to a new standard flat bottom 96-well plate and the 405 nm absorbance was measured using the Envision plate reader. The percentage of erythrocyte lysis was calculated using equation $\% Lysis= \frac{(Abs test-Abs vehicle)}{(Abs\max- Abs vehicle)} \times100$.

Enhancement of CDC lysis assay: A 50% (v/v) erythrocyte stock suspension was prepared as described above. This was diluted to 2.8% (v/v) working stock. 140 μL from the erythrocyte working stocks was added to each well of a V-bottom 96-well plate (Greiner, 651201). Two-fold serial dilutions of test compounds were prepared as a 10 × solution in the desired range. 20 μL from each test compound dilutions were added then 20 μL of Anti‑RBCs antibody (C_f_ = 40 or 80 μg/mL) was added to the corresponding test well. The plate was incubated at RT for 20 min then 20 μL from CS (C_f_ = 5%) was added to test wells. The following controls were included: (1) a positive control which contained Tween-20 (C_f_ = 1%), (2) a negative control well that contained PBS, and (3) a vehicle only well that contained PBS without erythrocytes to subtract the absorbance of the vehicle. The plate was then incubated at 37 °C for 1 h and then centrifuged (2500 rpm, 5 min) to pellet any un-lysed erythrocyte. 100 μL from the supernatant was transferred to a new standard flat-bottom 96‑well plate and the 405 nm absorbance was measured using the Envision plate reader. The percentage of erythrocyte lysis was calculated using $\% Lysis= \frac{(Abs test-Abs vehicle)}{(Abs\max- Abs vehicle)} \times100$.

**4. Appendix**

HPLC traces and mass spectra for all peptides.

HPLC traces and mass spectra for synthesised hits from enriched screen were ran by method B (see Analysis and purification)

CP-02


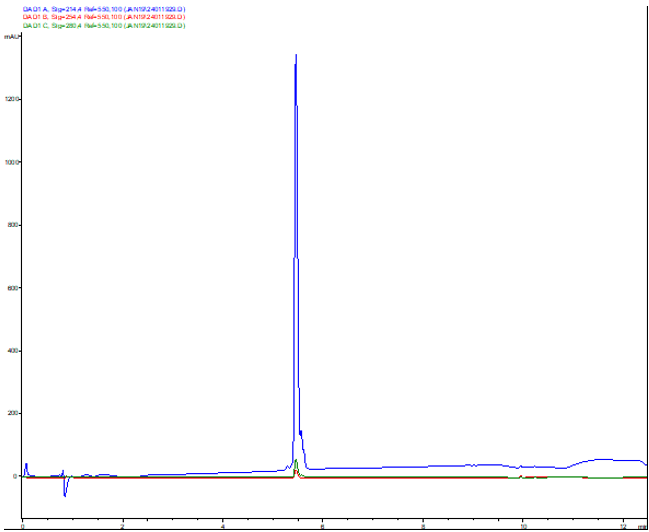

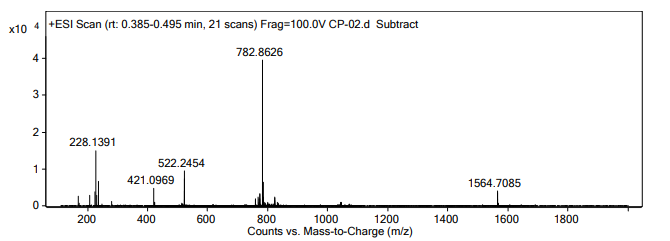


CP-03


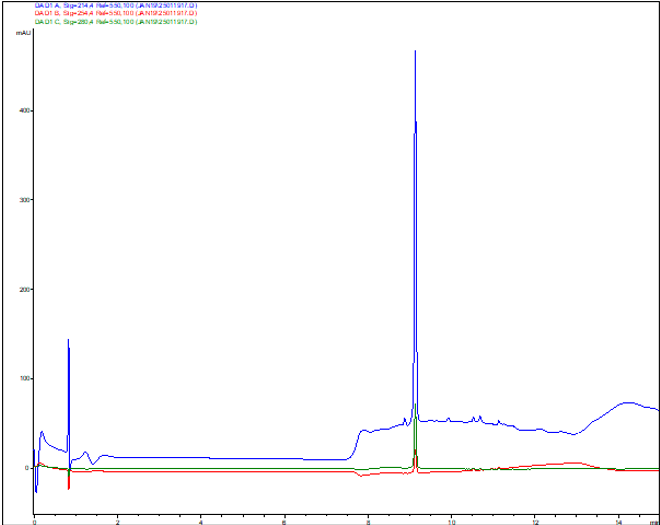

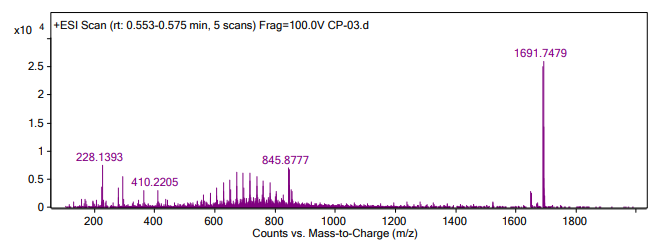


CP-04


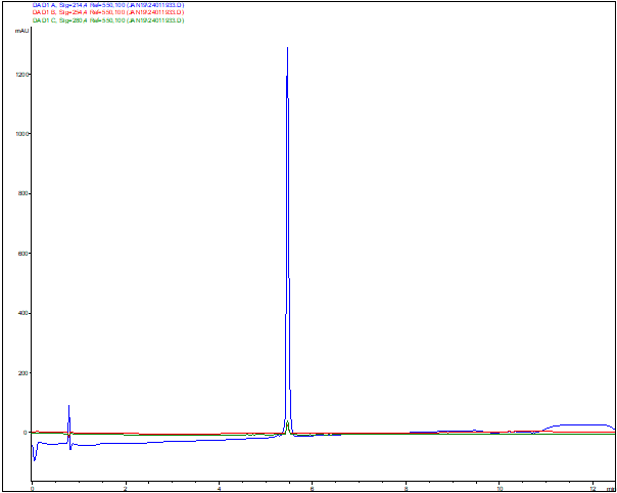

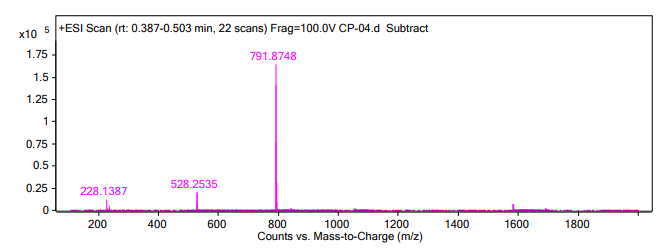


CP-06


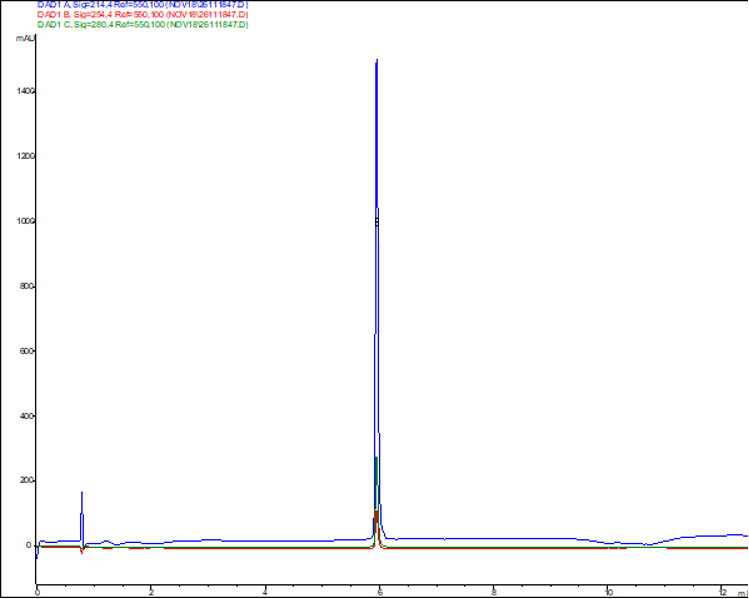

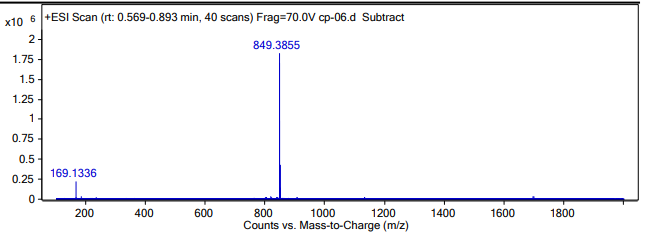


CP-13


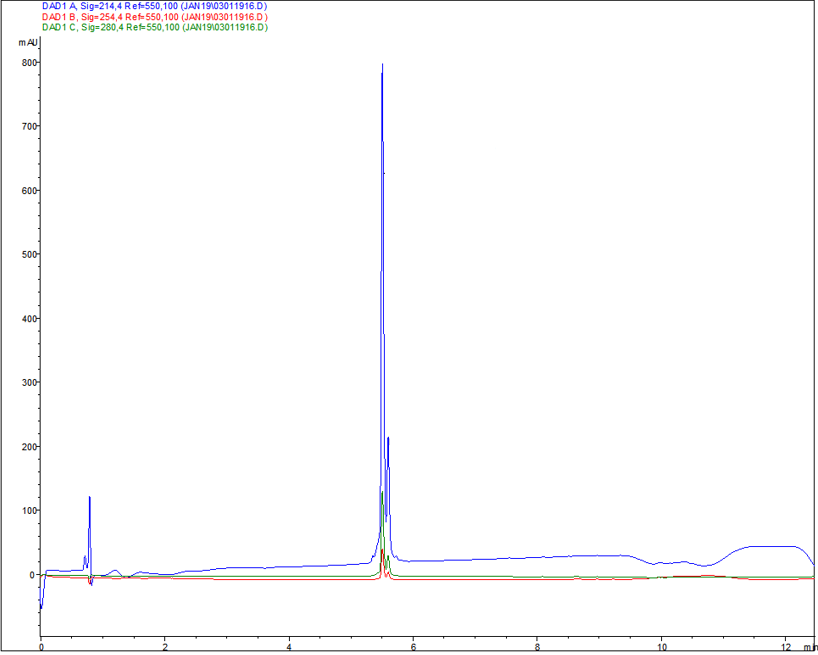

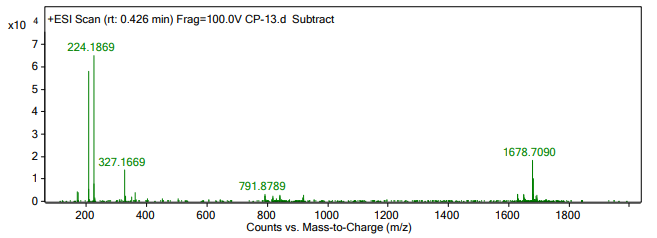


All CP-06 analogues other than C-18 tailed were ran by method A (see Analysis and purification)

CP-06_scrmb_

_
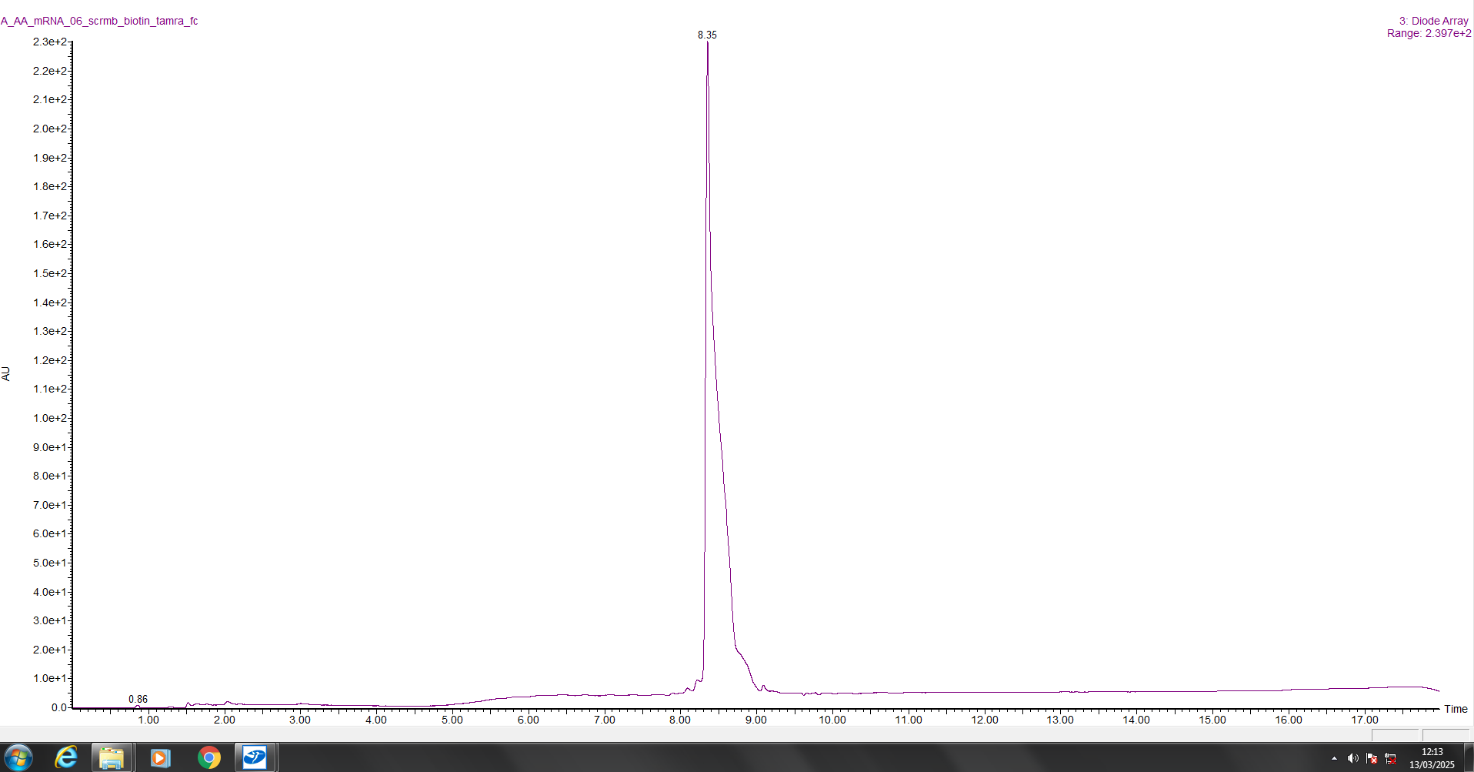
_


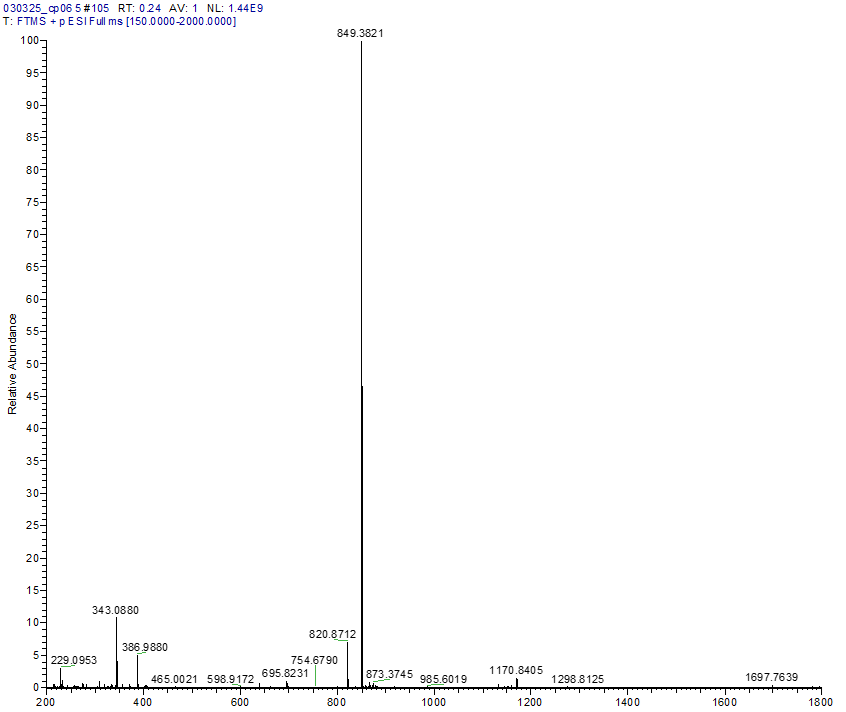


CP-06-linear


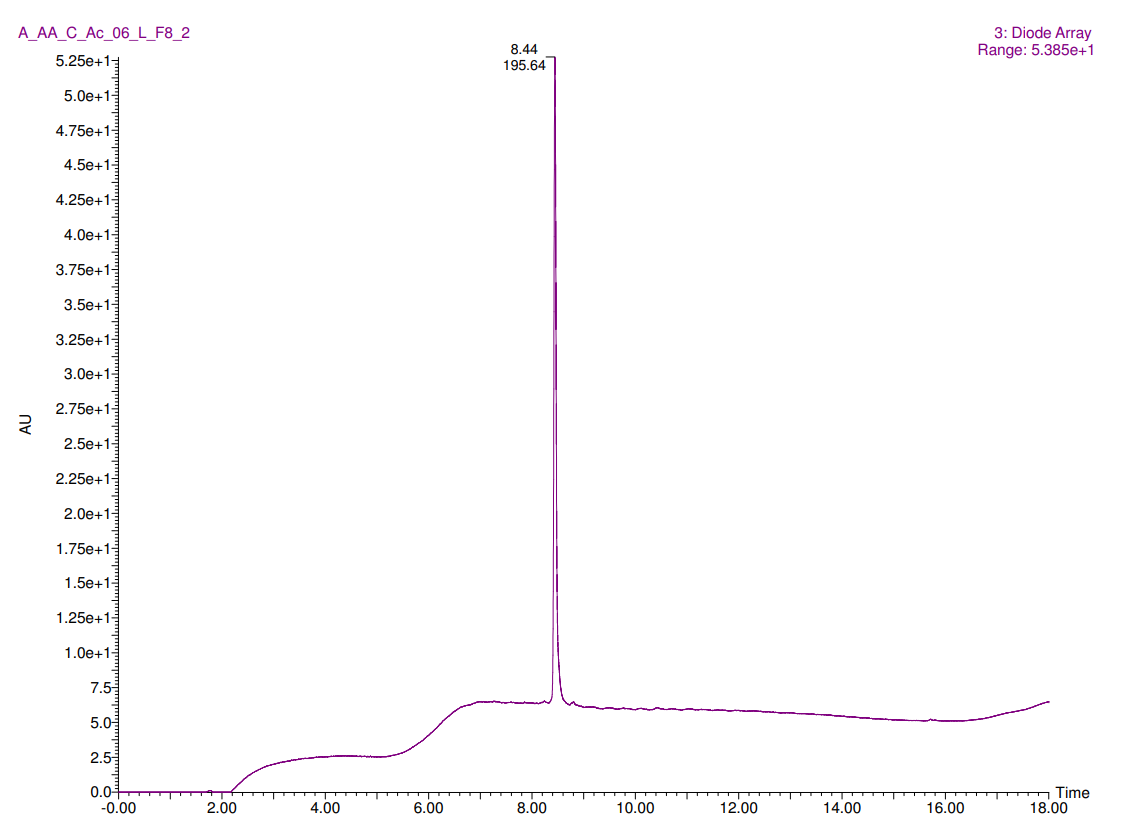


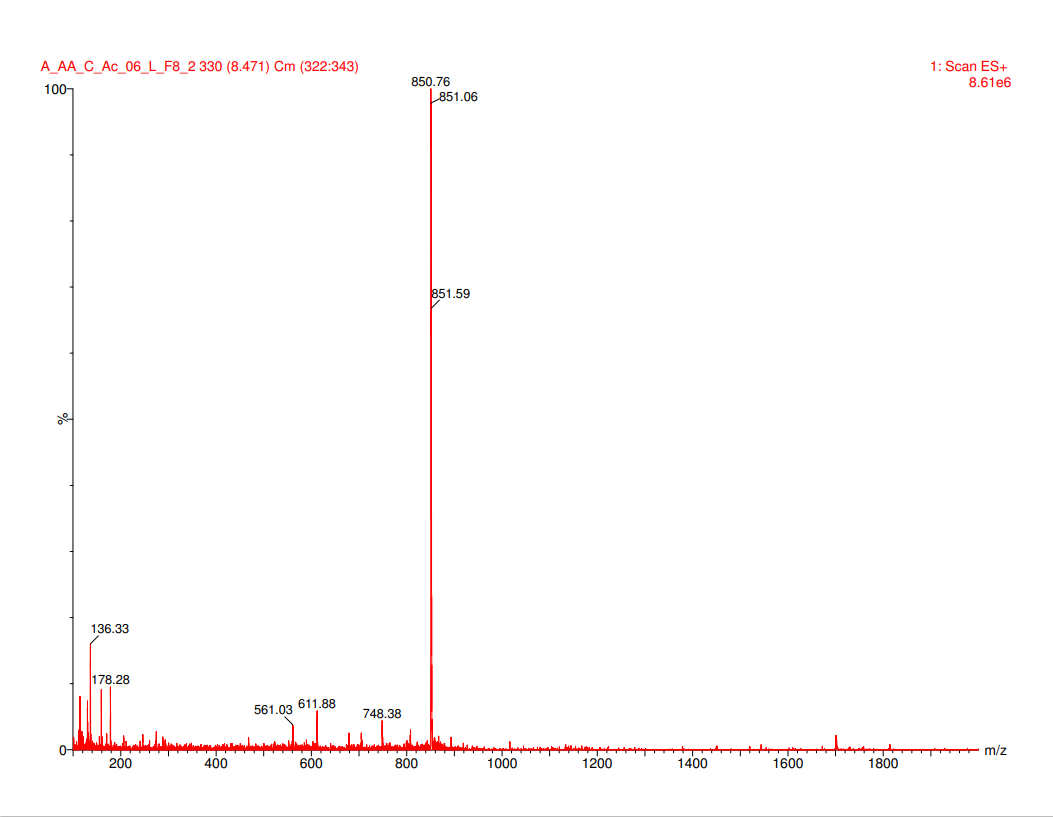


Y1A


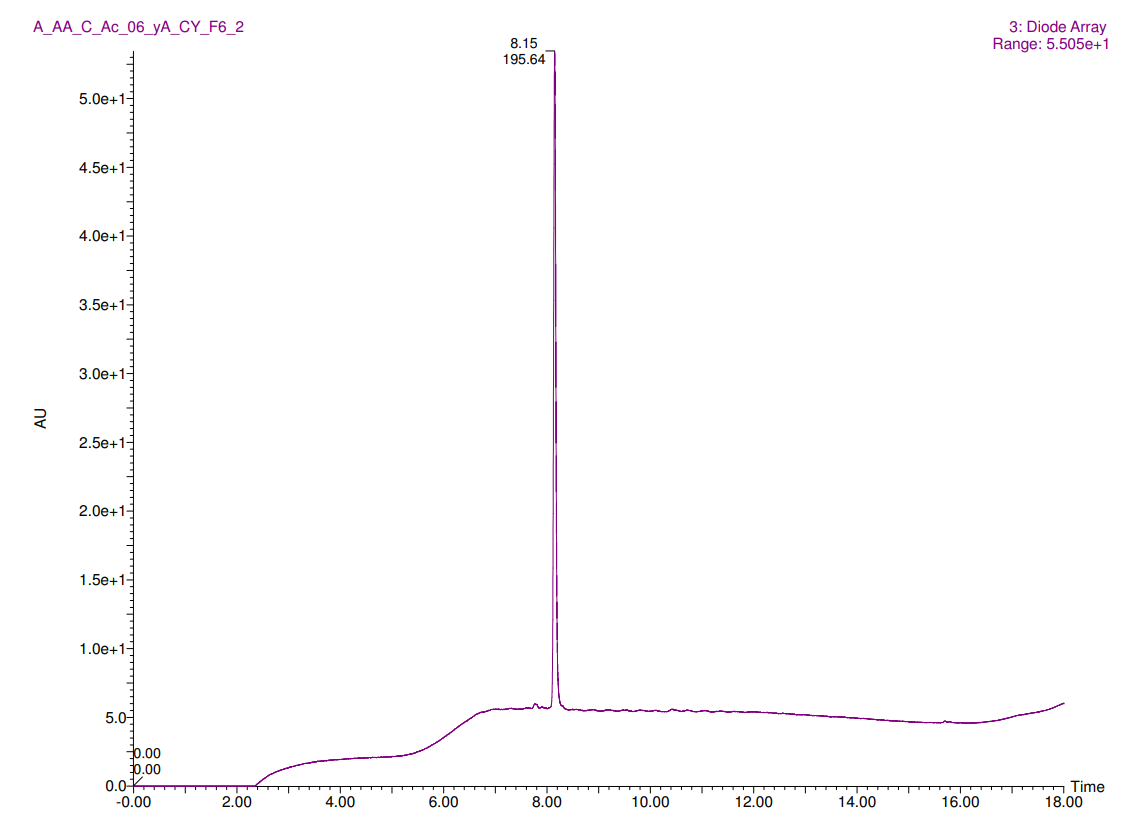


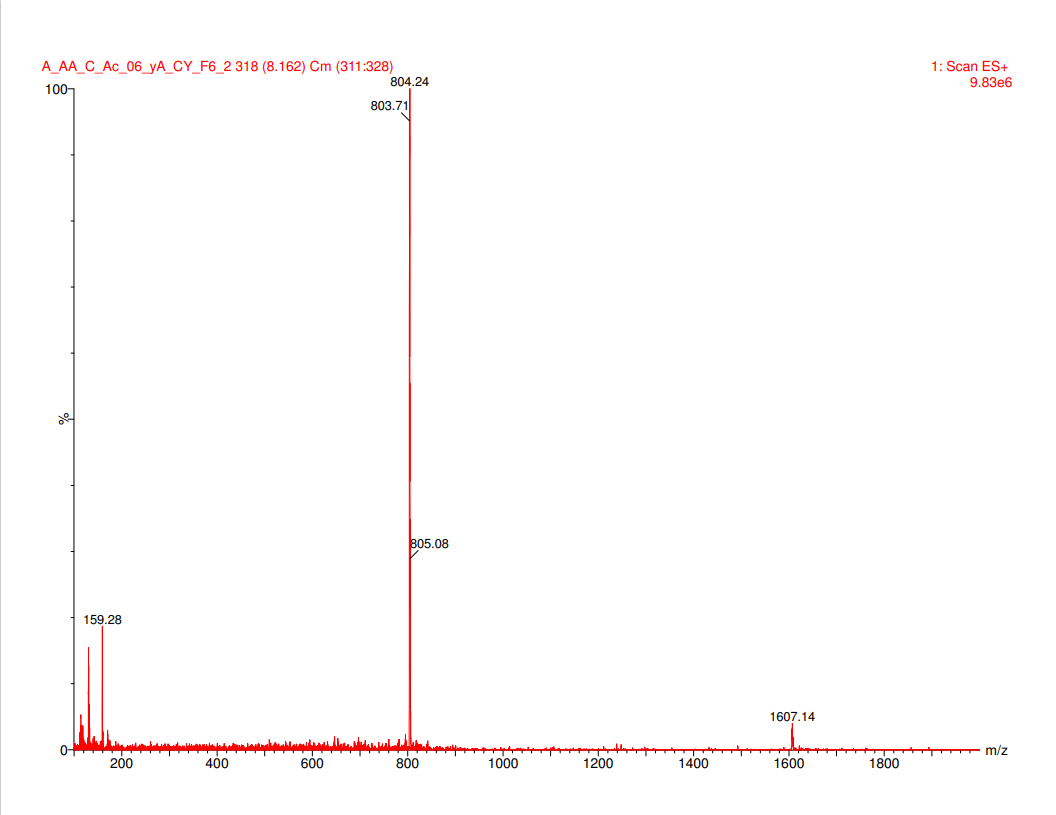


S2A


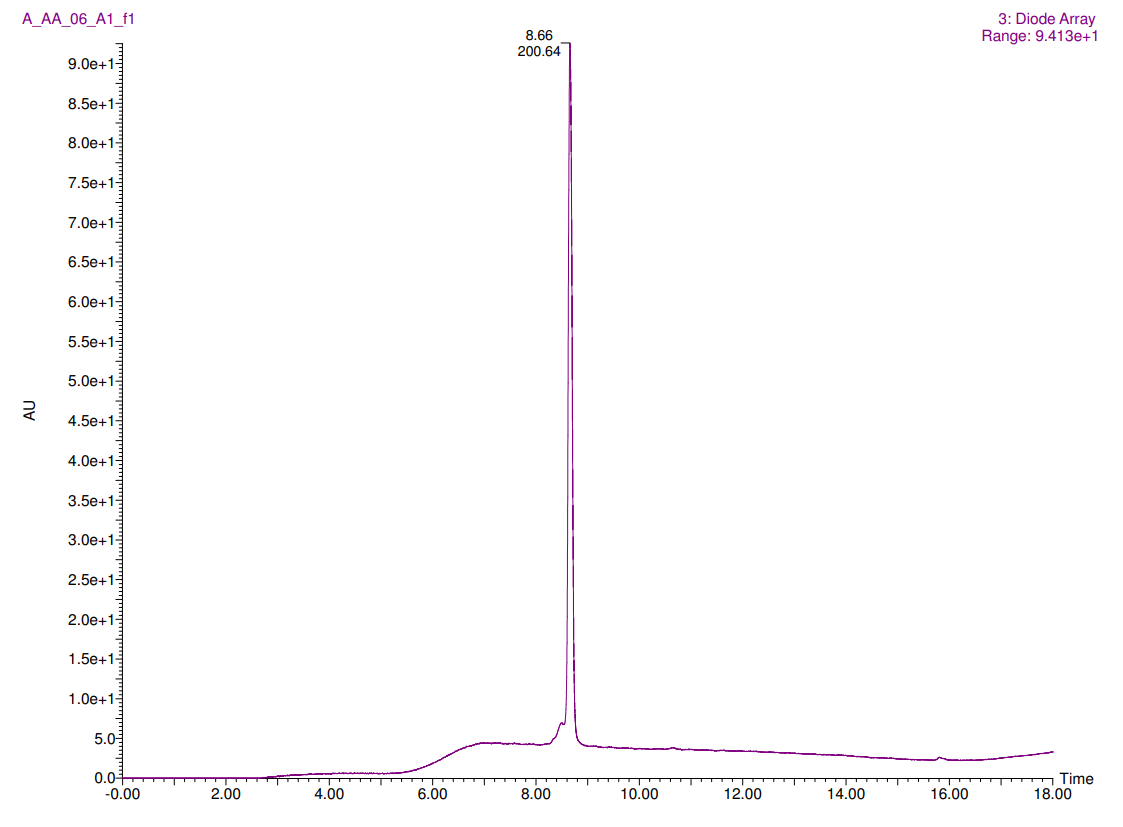


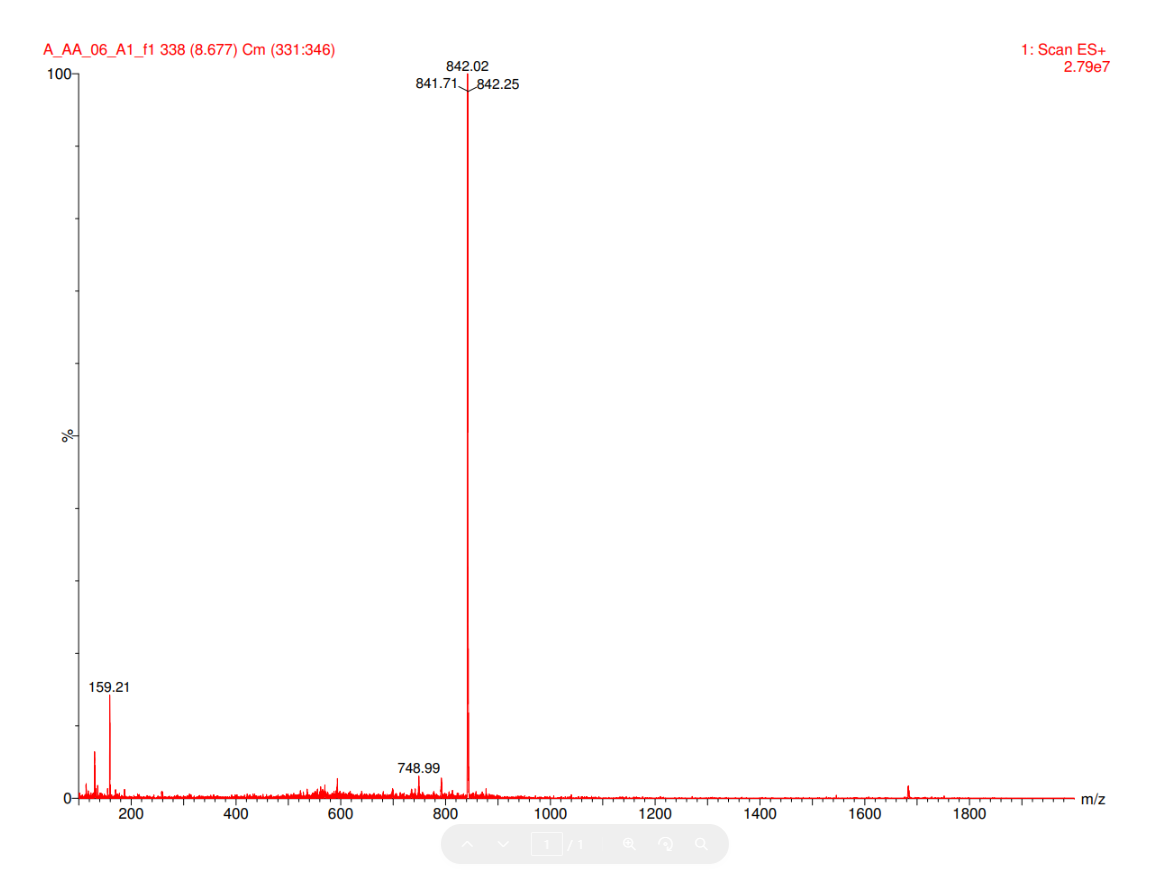


W3A


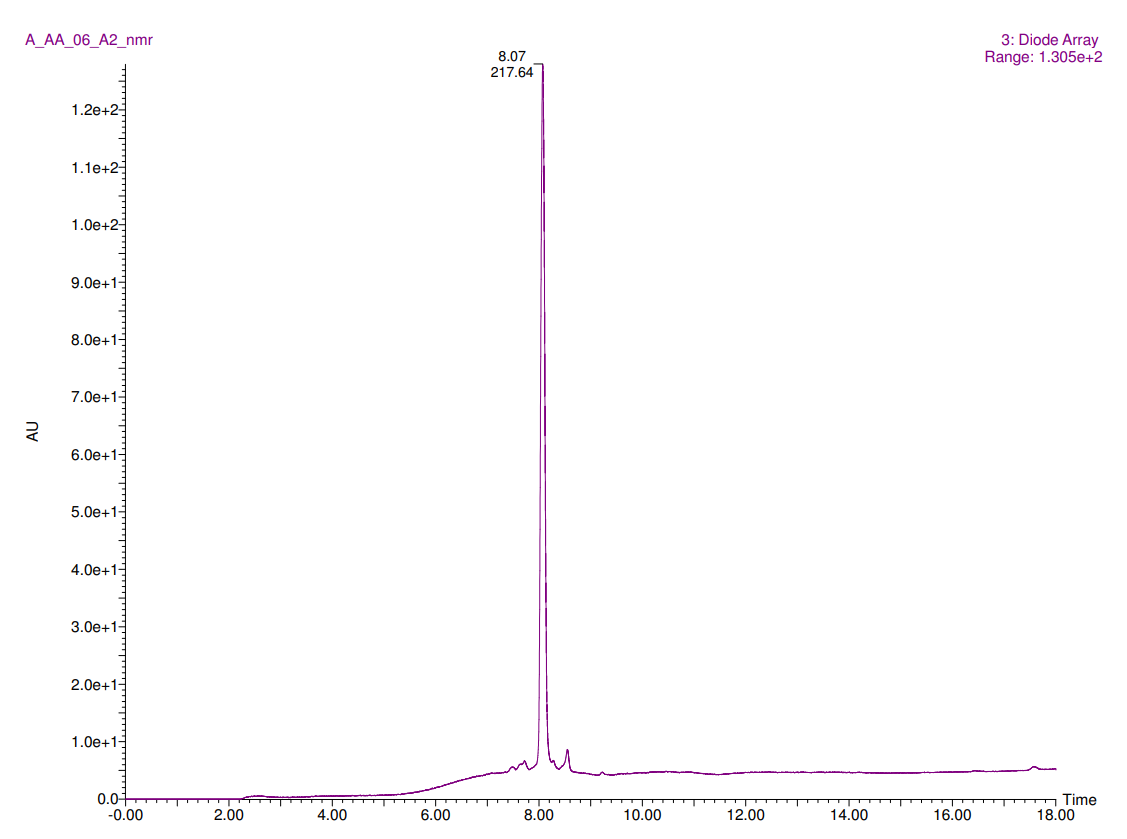


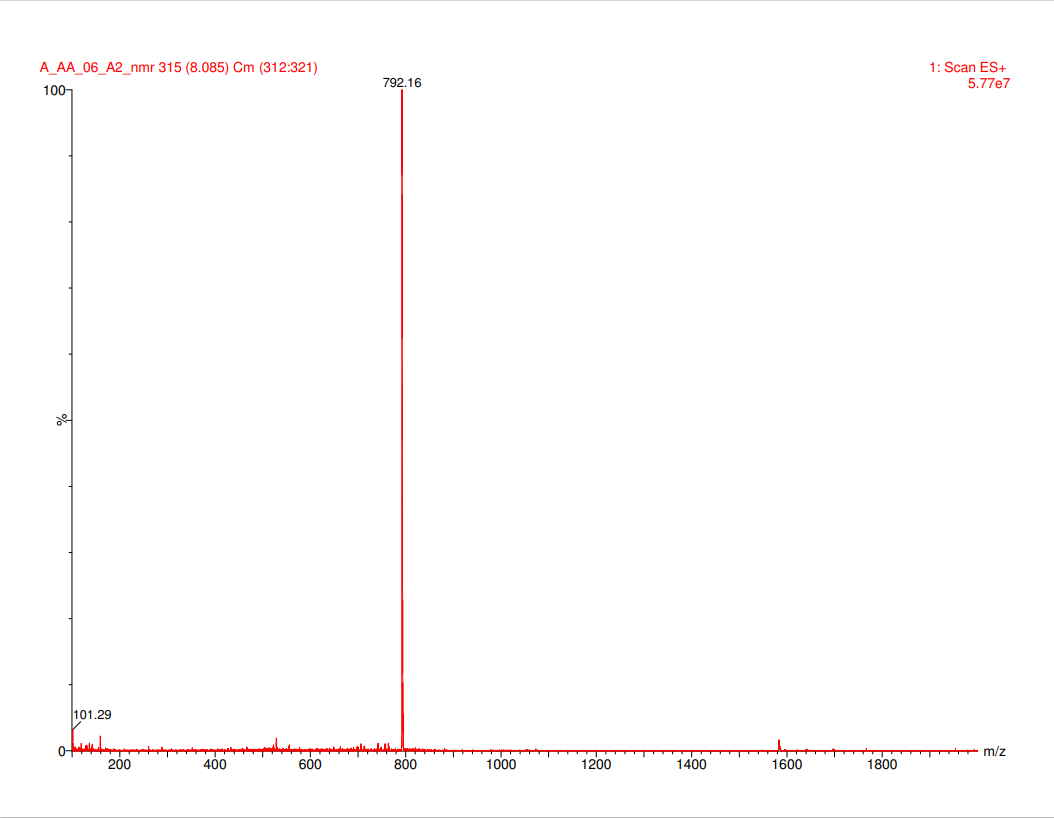


T4A


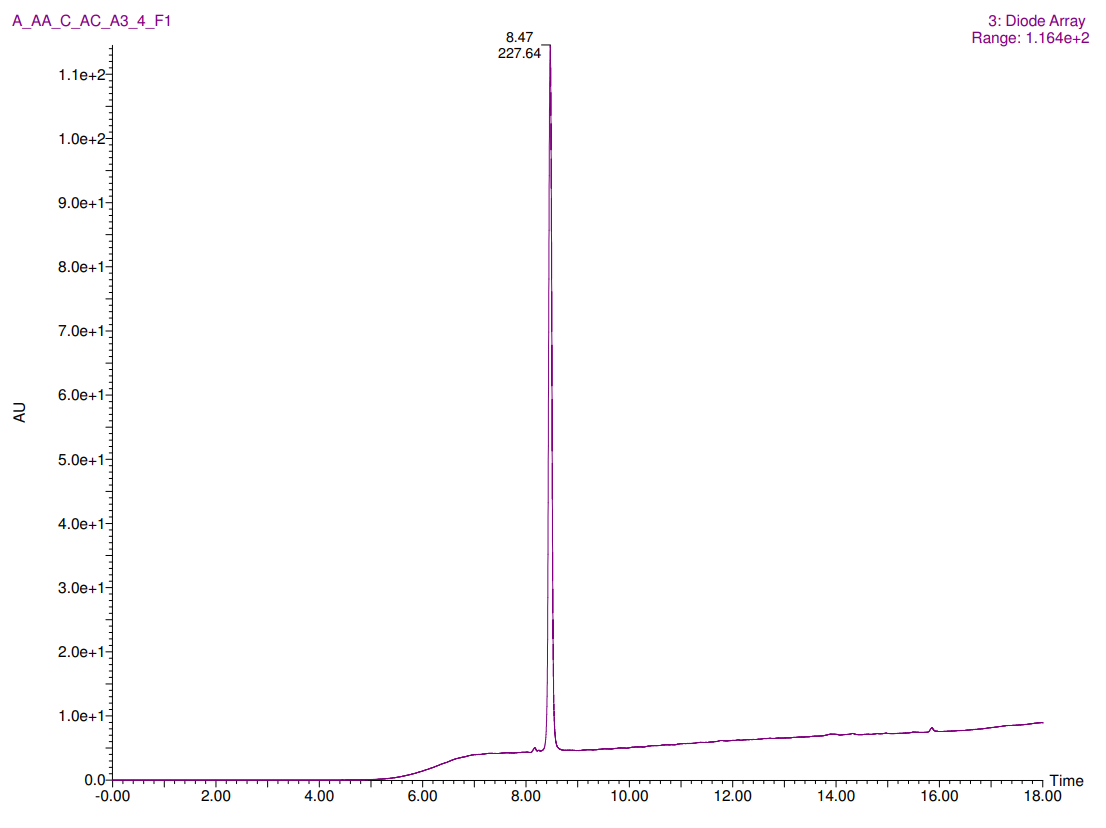


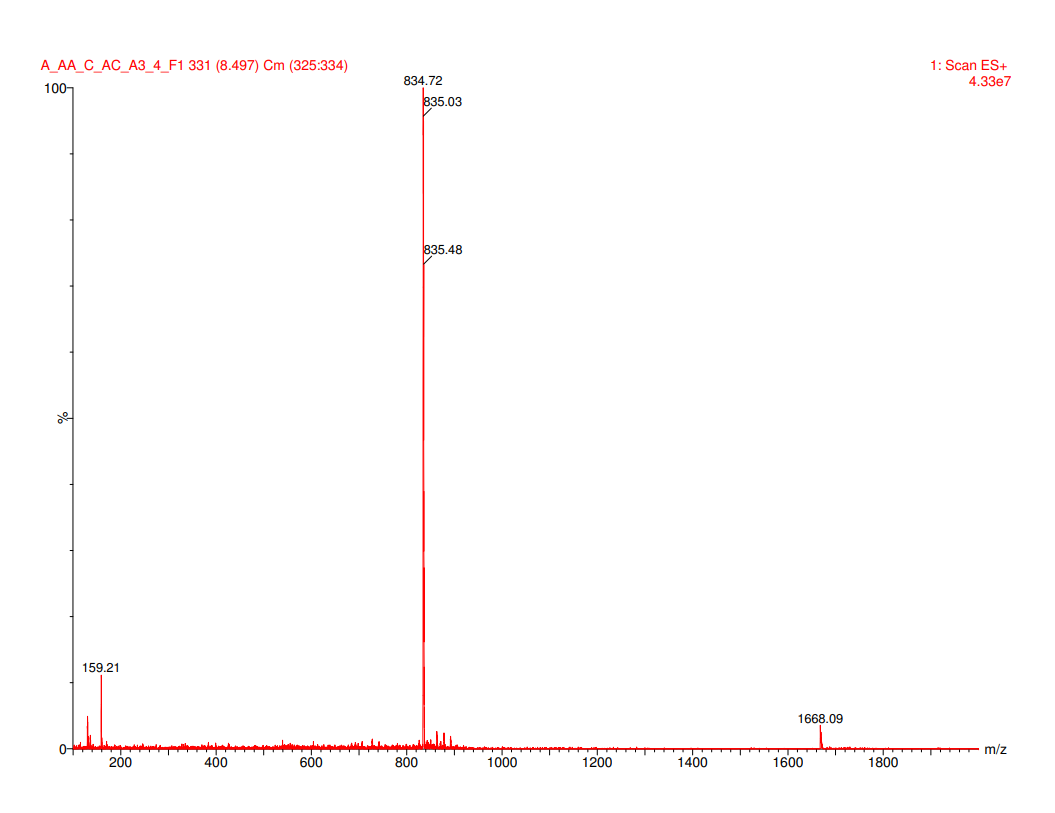


W5A


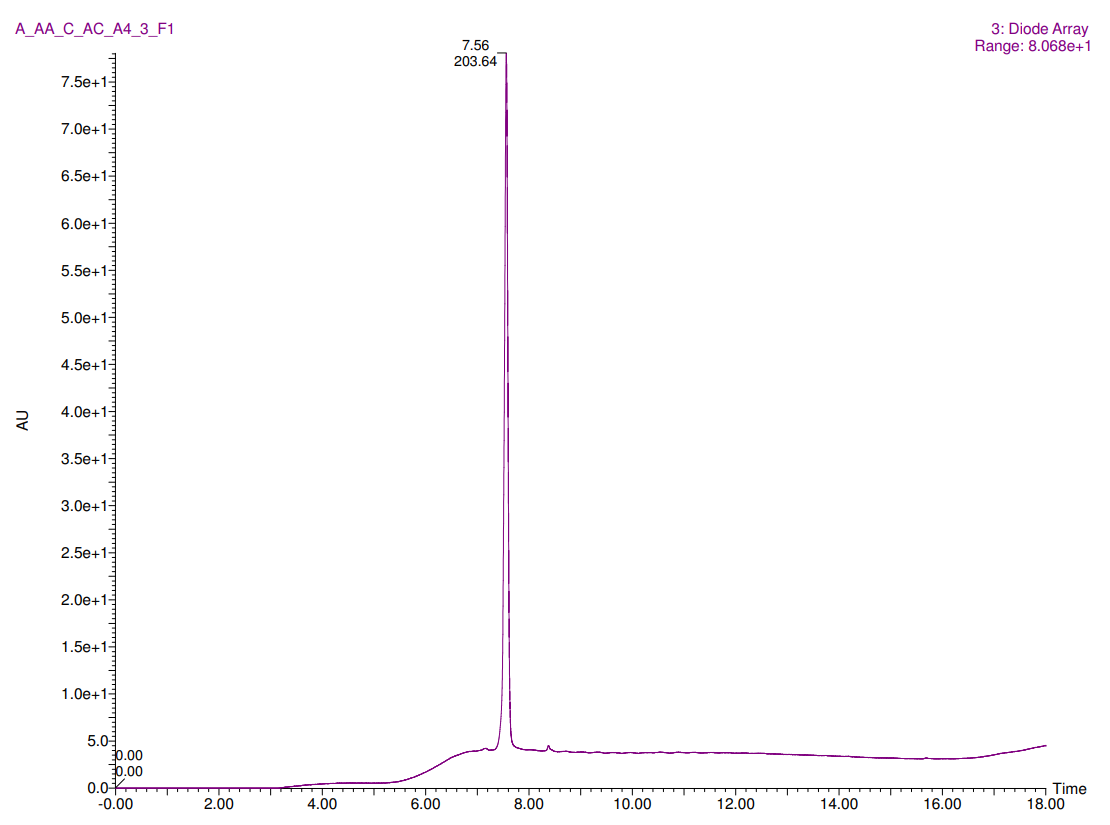


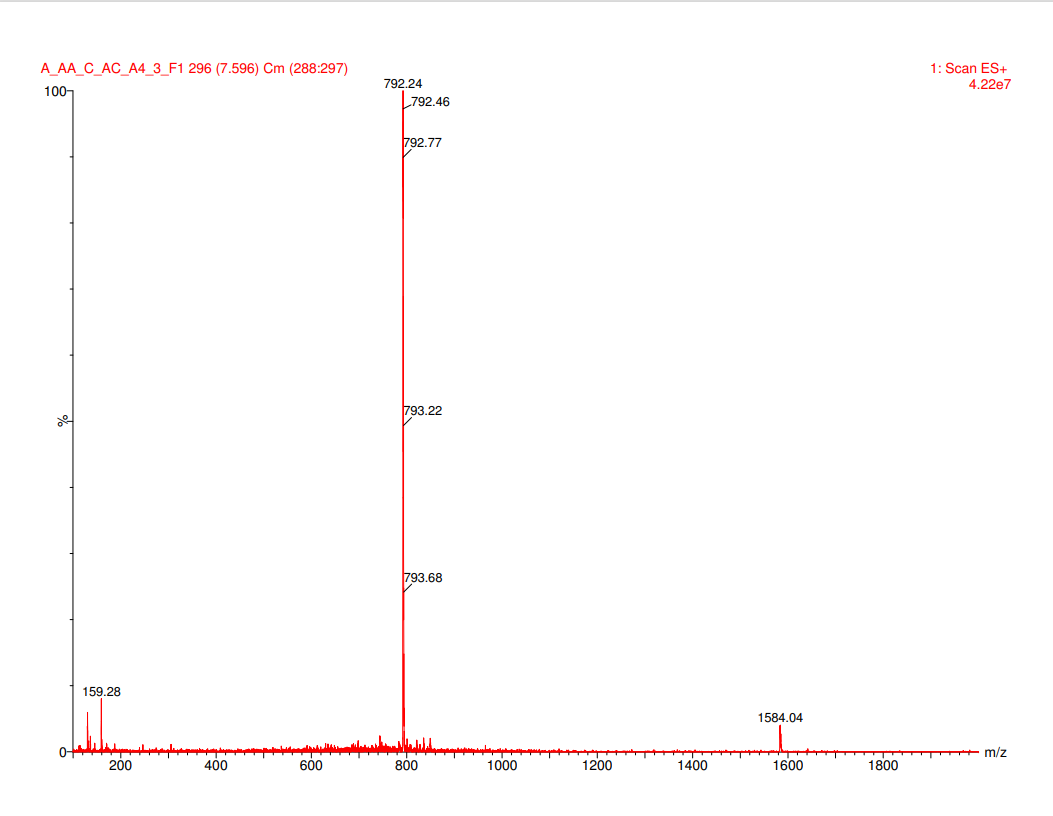


G6A


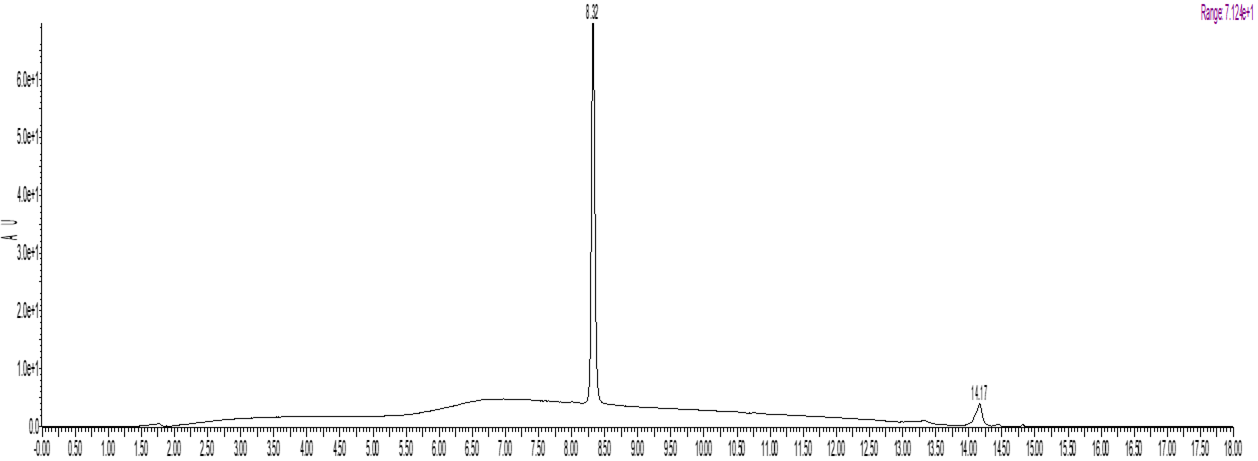


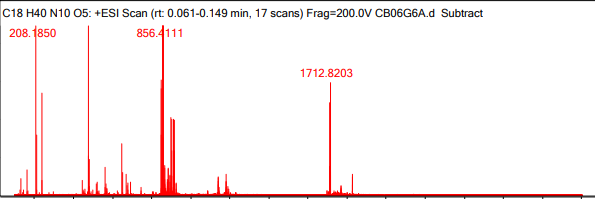


N7A


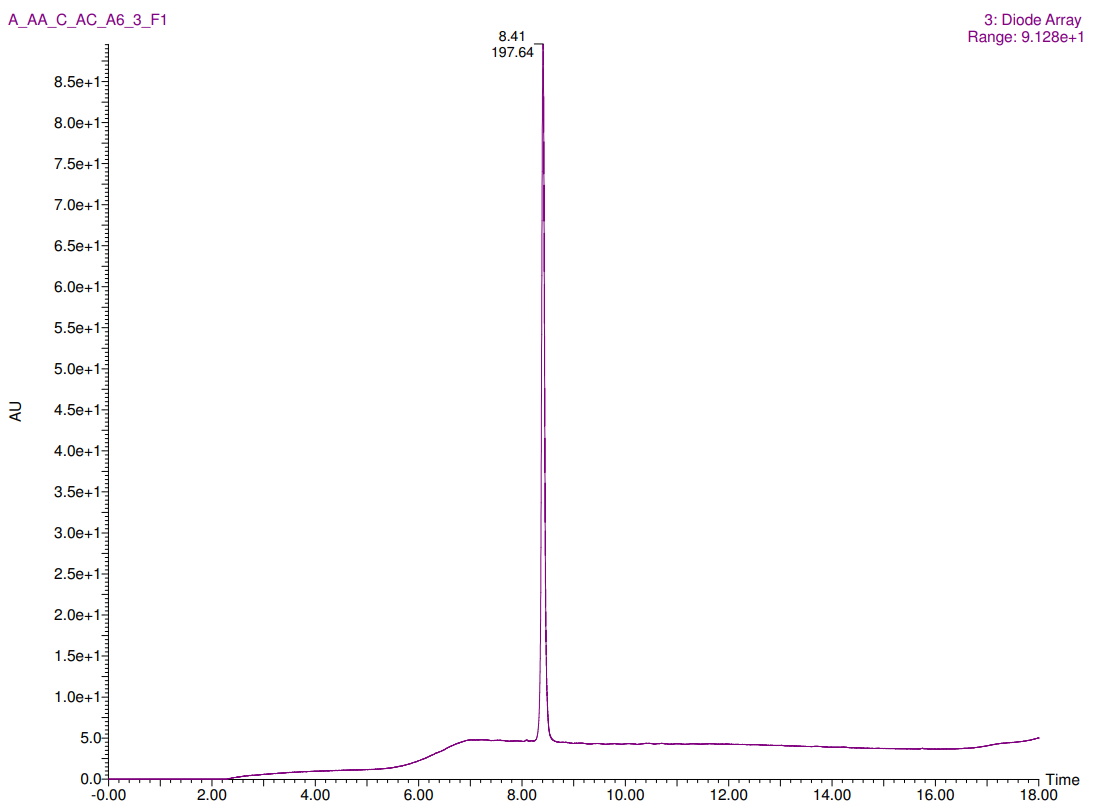


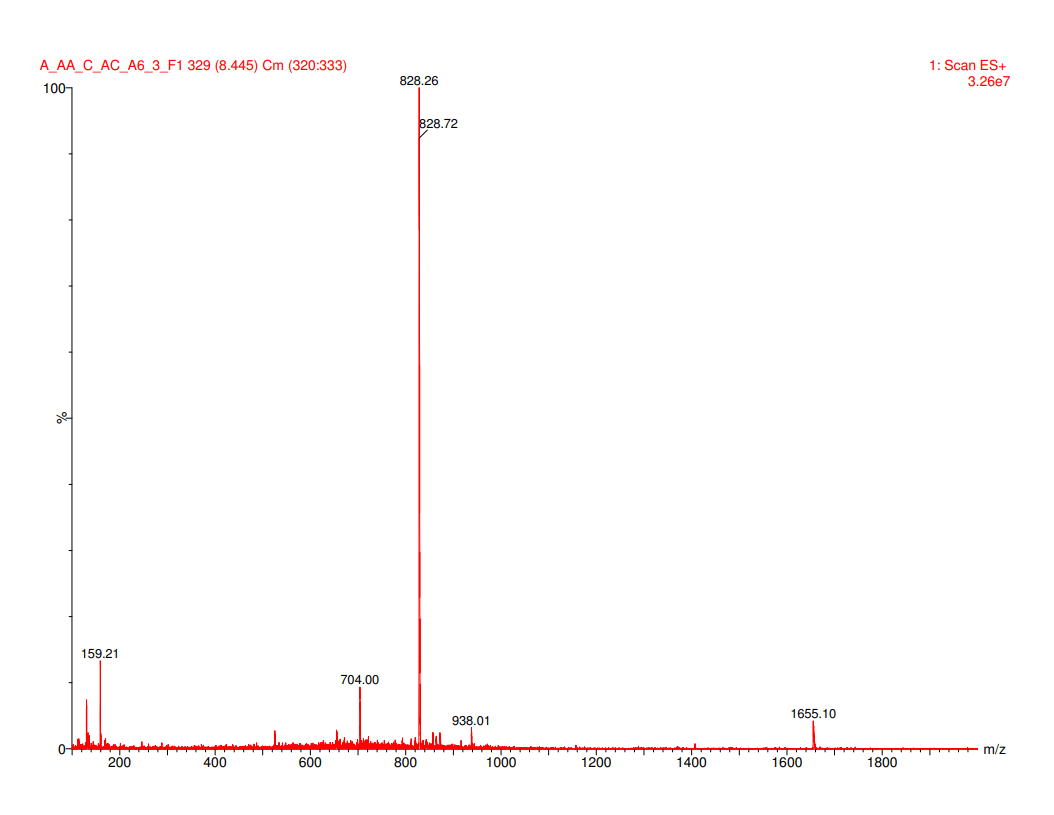


R8A


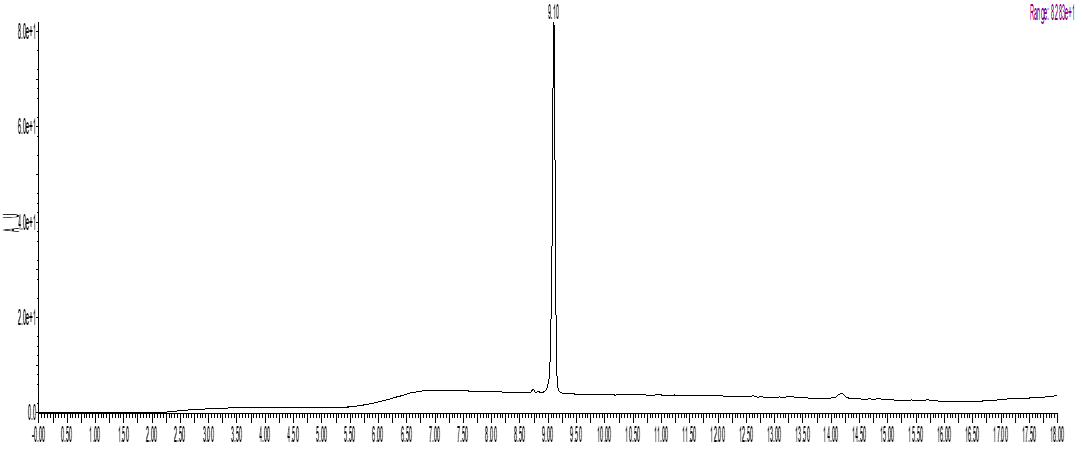


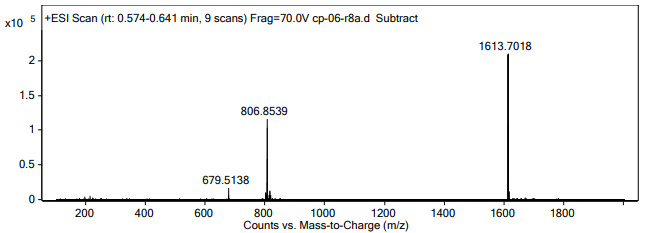


S9A


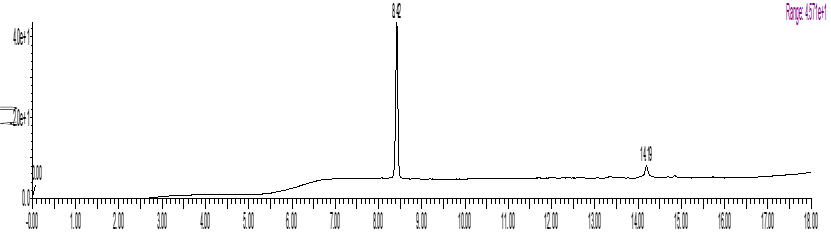


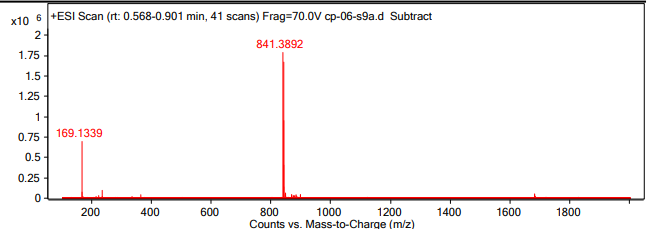


S10A


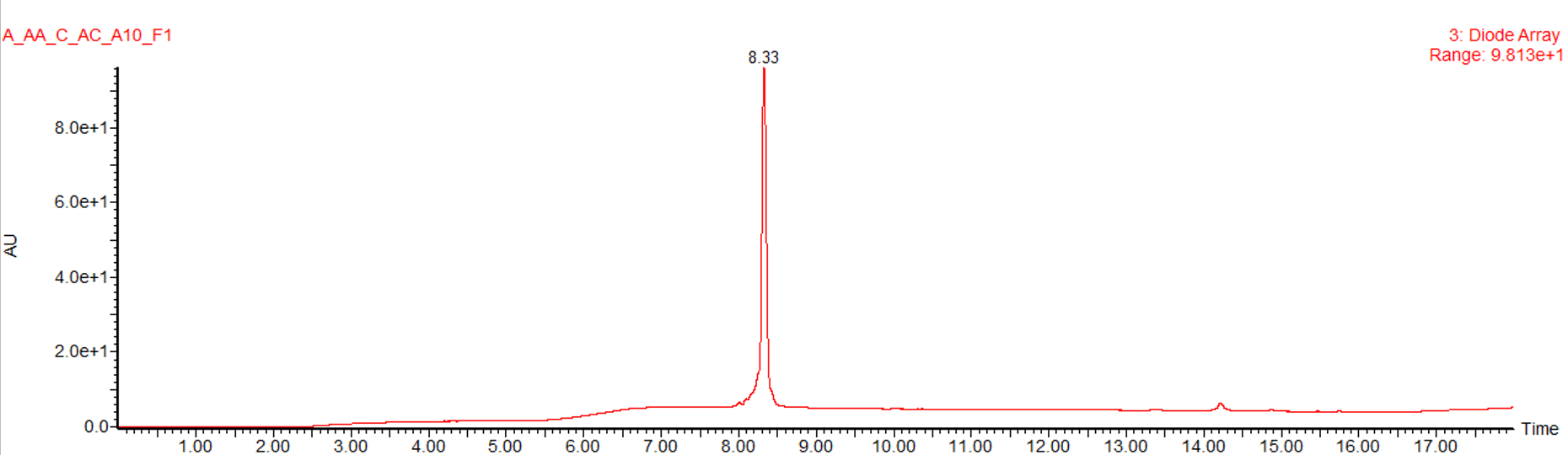


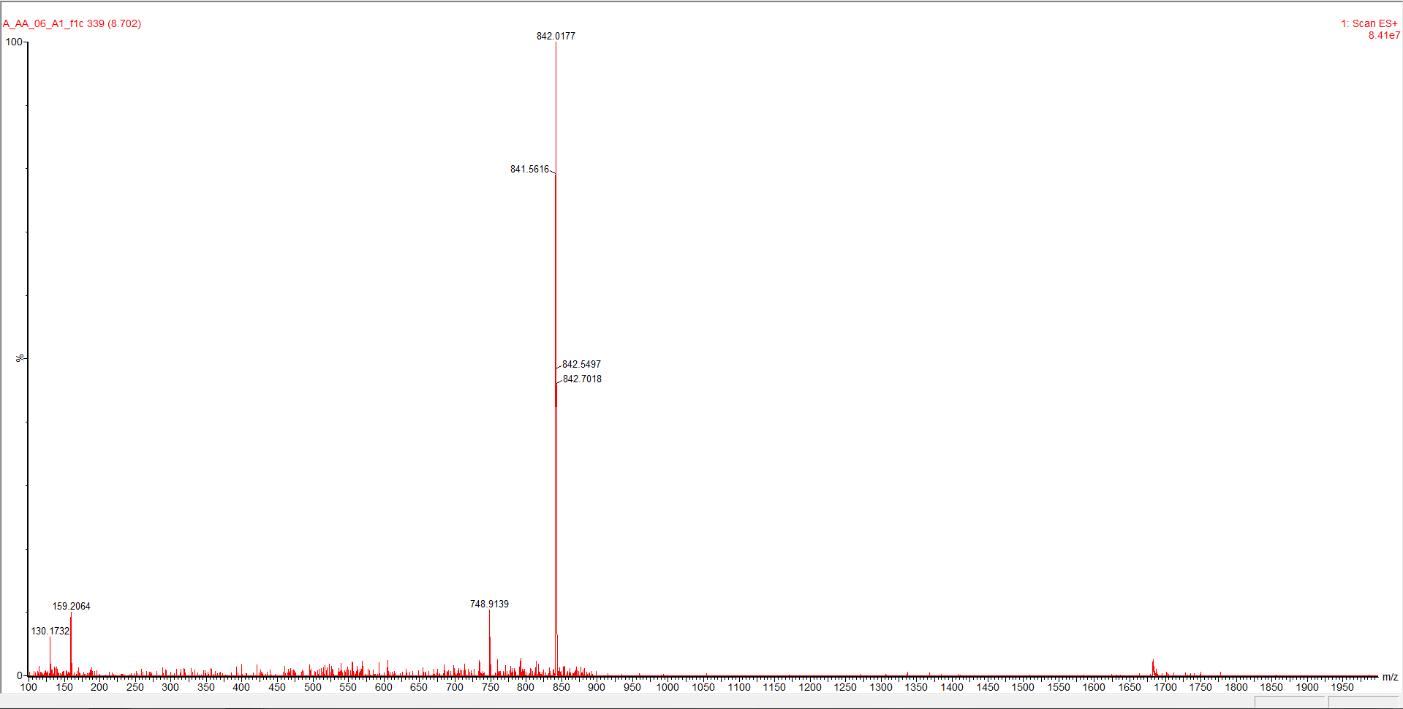


V11A


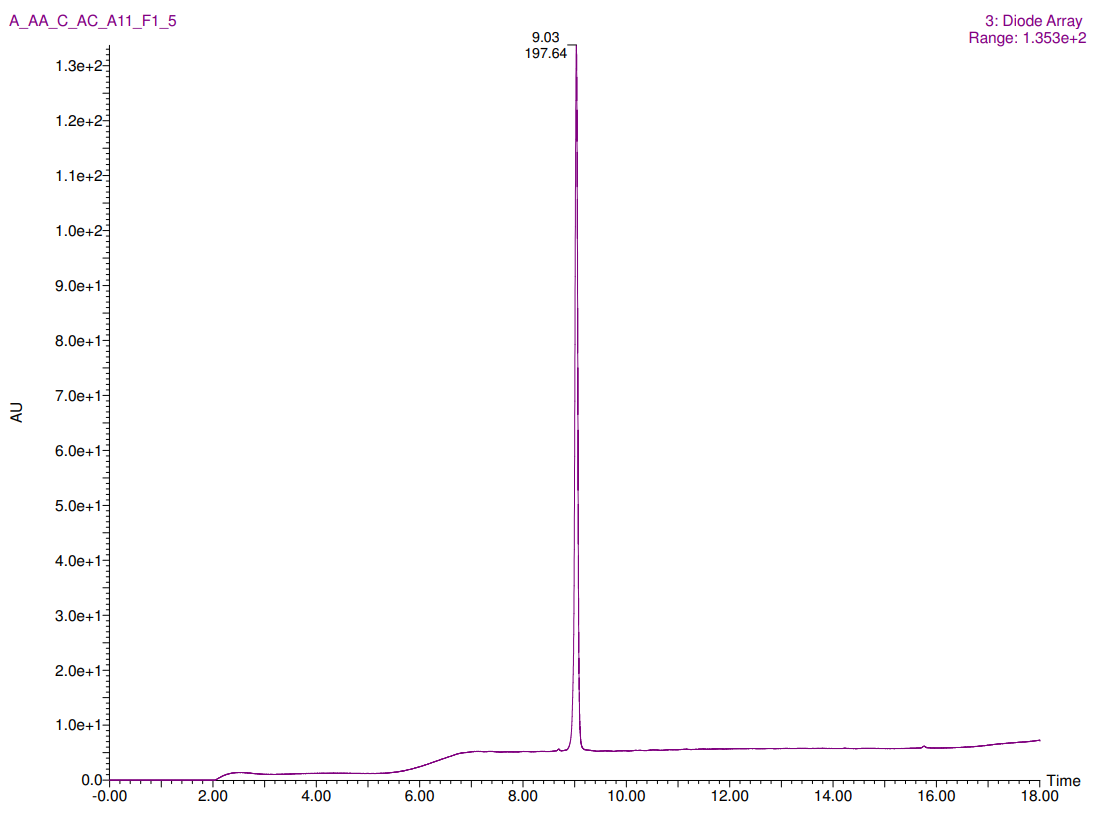


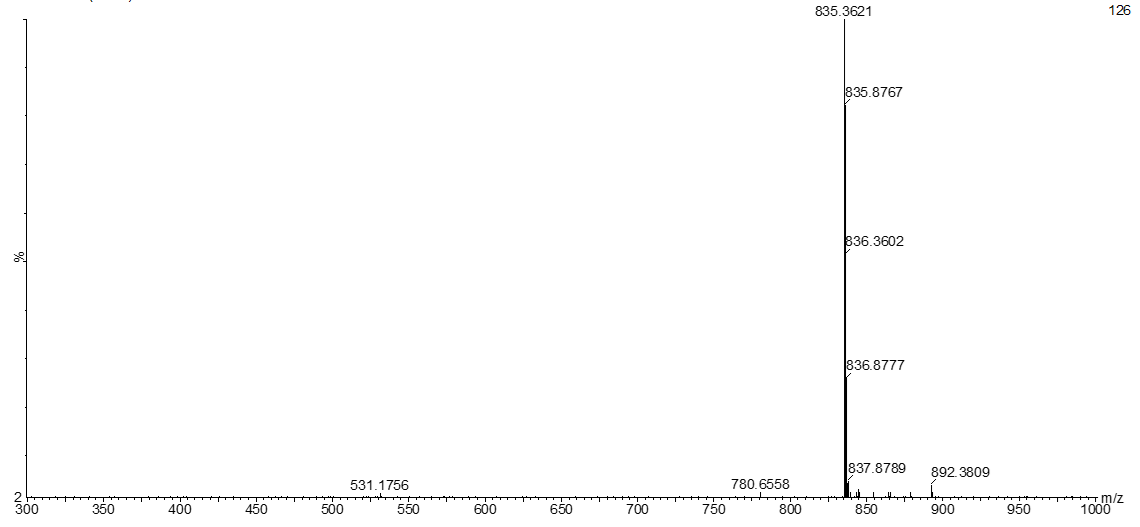


R12A


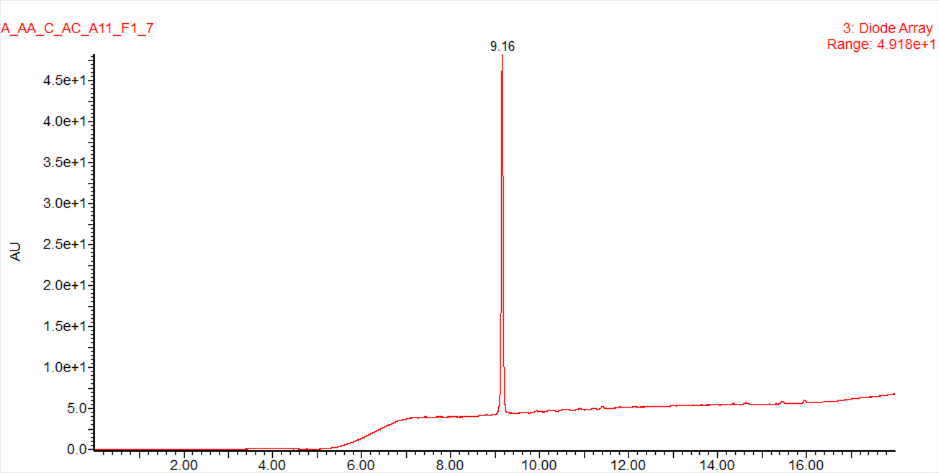


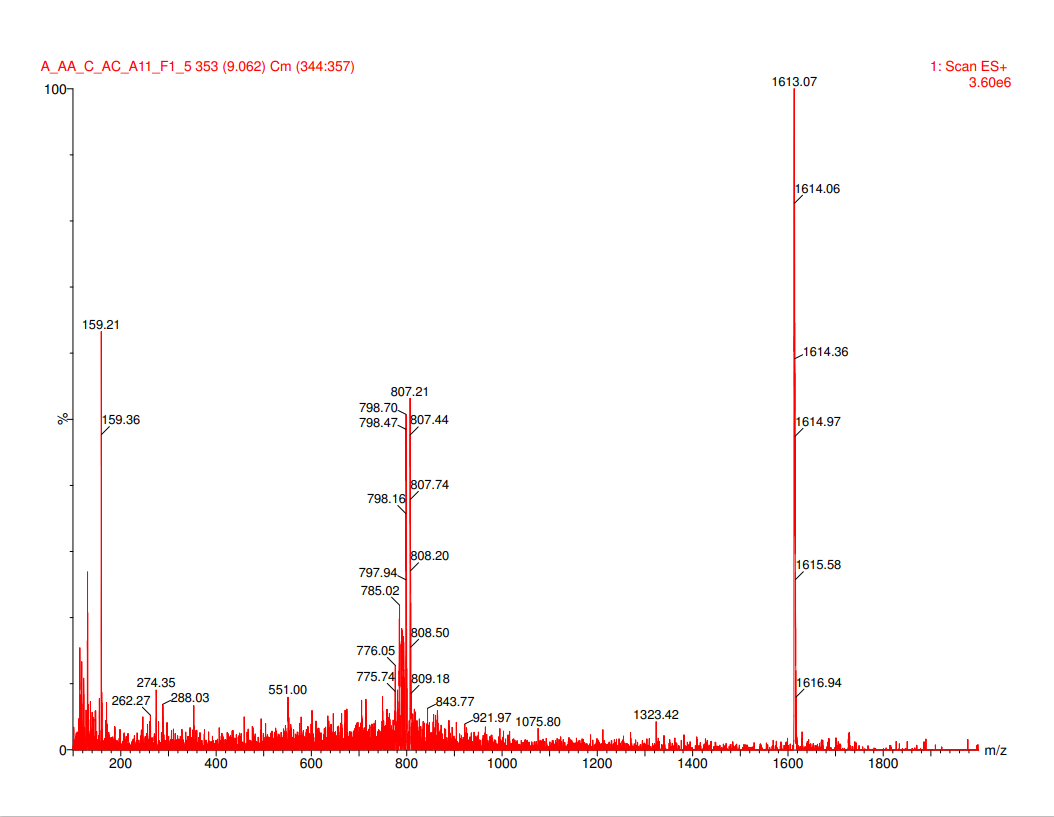


G13A


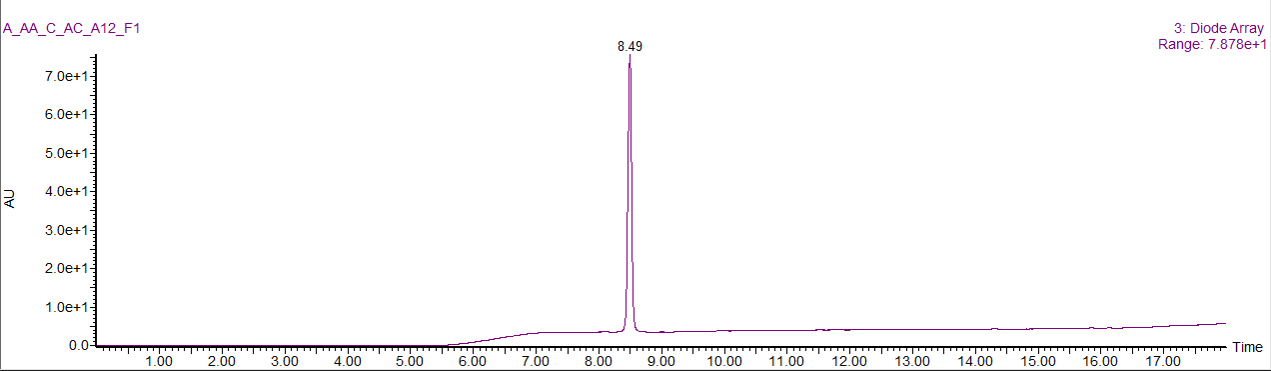


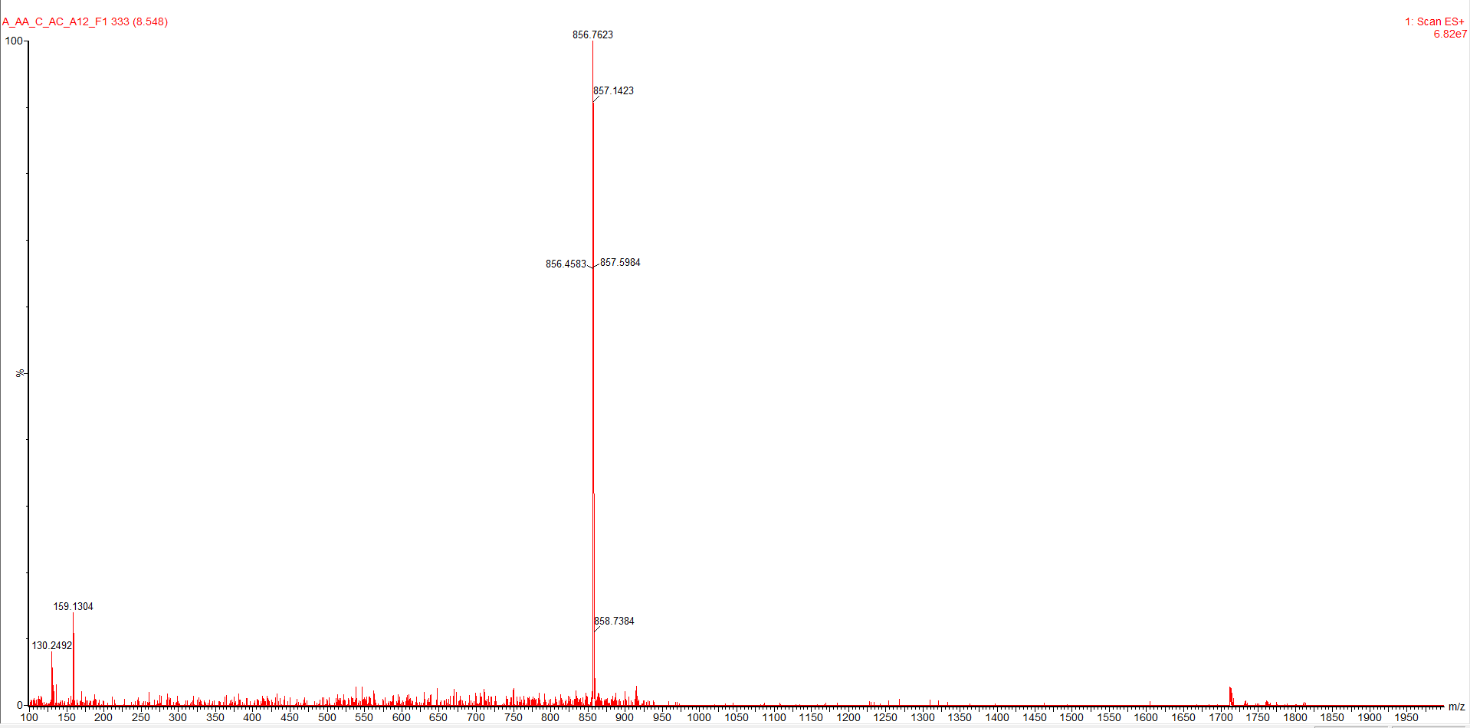


CP-06-GKG


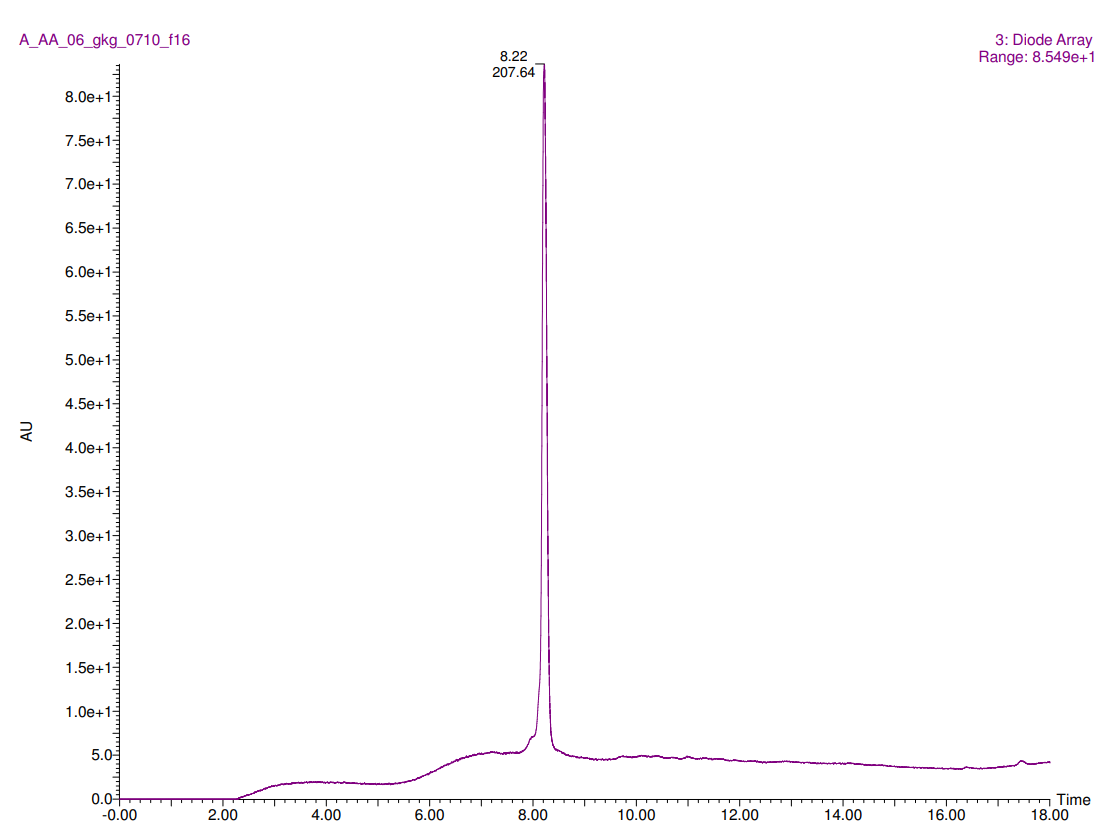


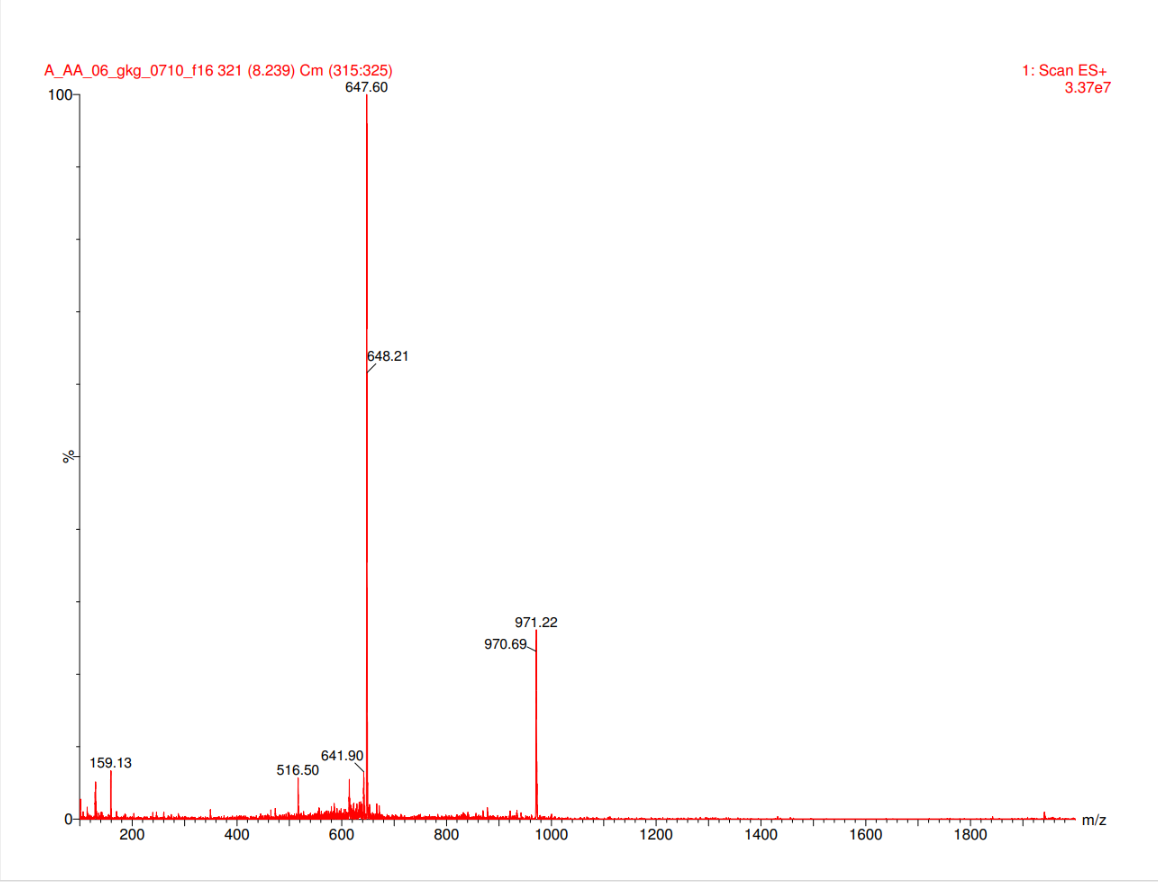


CP-06-GSGSGSK


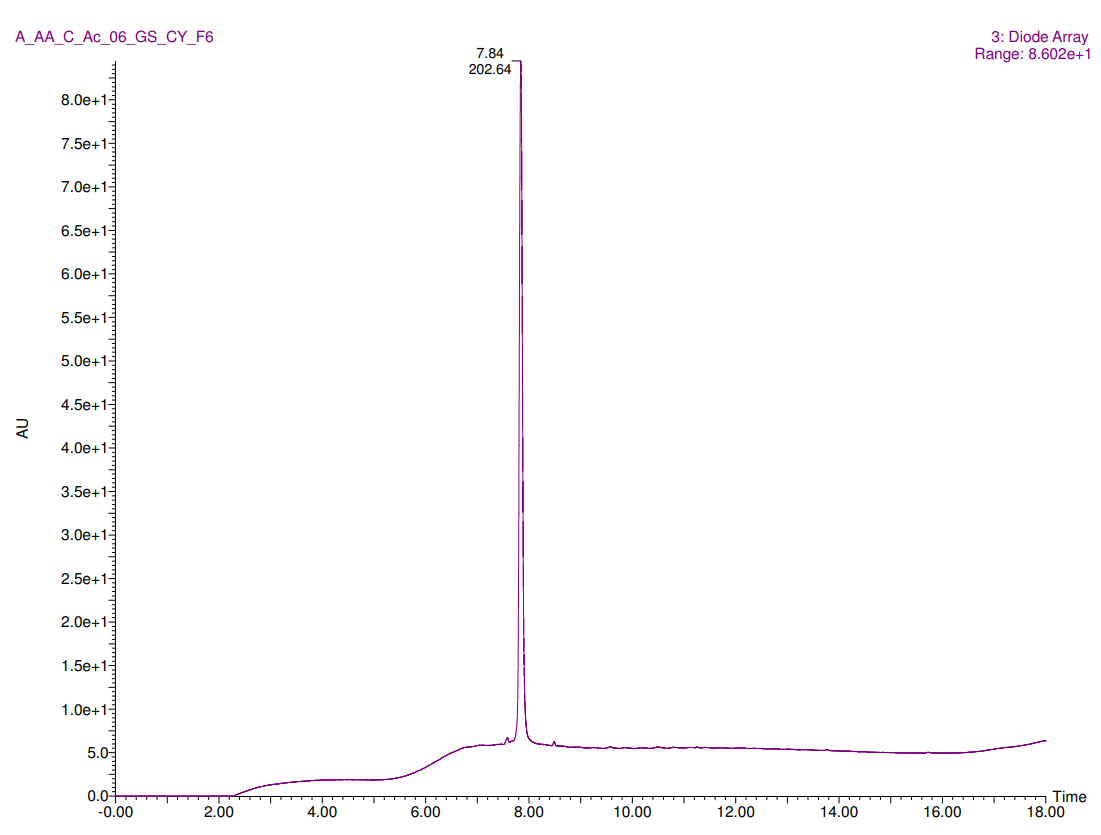


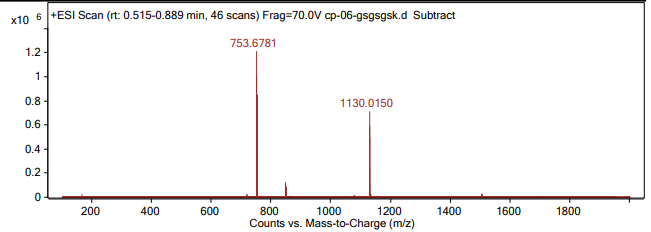


CP-06-dimer


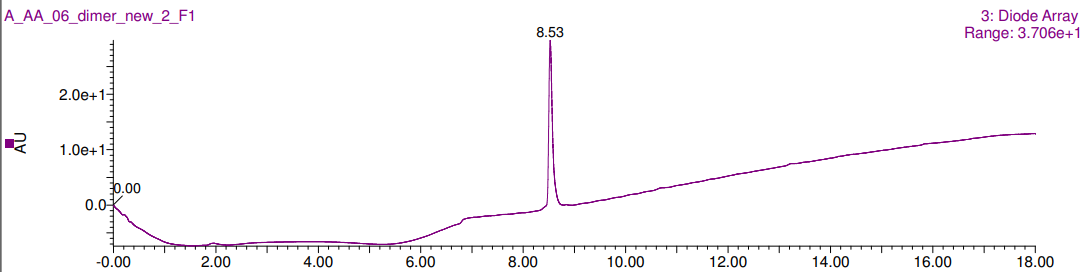


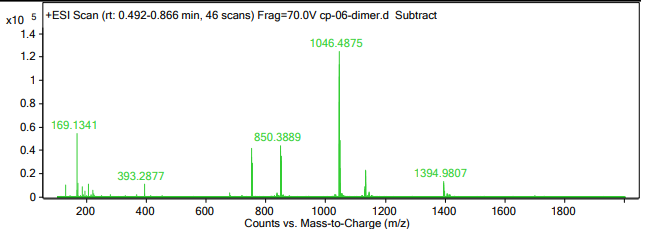


CP-06_C18_ analogues were ran by method C (see Analysis and purification)

CP-06_C18_


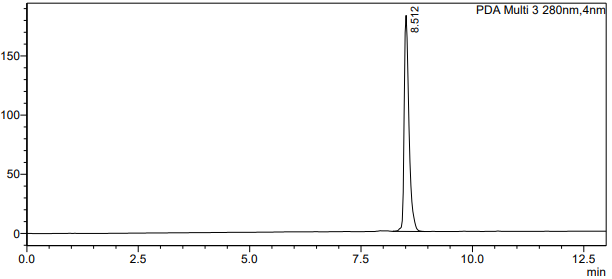


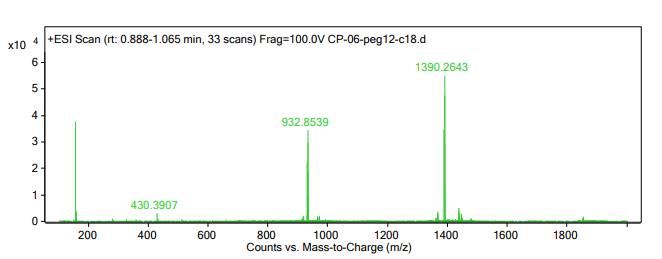


CP-06_scrmb_-_C18_


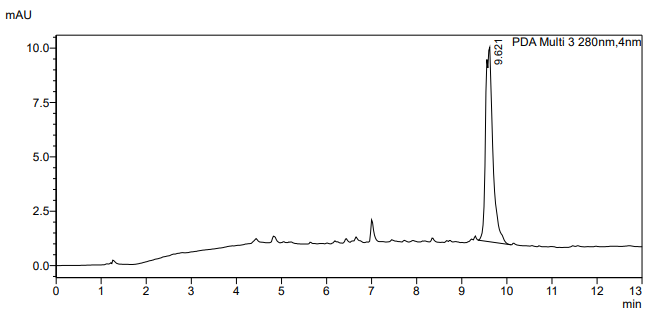


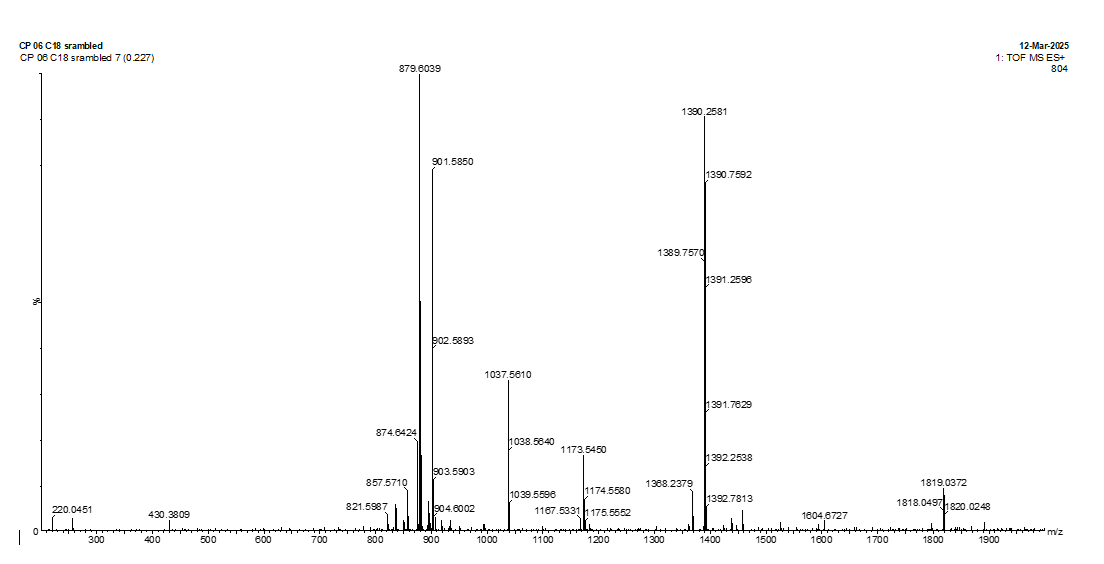


Table S3: A table of the found masses from HRMS experiments for initial peptides hits synthesised and those presented in figure 5.

| Peptide | MW | Expected | Charge | Found | Error (PPM) |
| --- | --- | --- | --- | --- | --- |
| CP-02 | 1563.7001 | 782.8573 | 2 | 782.8626 | 6.77 |
| CP-03 | 1689.7358 | 1690.7431 | 1 | 1690.7425 | 0.35 |
| CP-04 | 1581.7259 | 791.8703 | 2 | 791.8748 | 5.68 |
| CP-06 | 1696.7529 | 849.3837 | 2 | 849.3855 | 2.12 |
| CP-06_scrmb_ | 1696.7529 | 849.3837 | 2 | 849.3821 | 1.88 |
| CP-13 | 1677.6994 | 1678.7067 | 1 | 1678.7090 | 1.37 |
| CP-06_dimer_ | 4179.918 | 1046.2376 | 4 | 1046.2385 | 0.86 |
| CP-06_C18_ | 2777.4925 | 1390.2552 | 2 | 1390.2643 | 6.55 |
| CP-06_scrmb-C18_ | 2777.4925 | 1390.2552 | 2 | 1390.2581 | 2.09 |

Table S4: A table containing of the found masses for the Ala scan peptides presented in Fig 3B.

| **Peptide** | **M_exact_ (Calc)**  **[Da]** | **Mw (Calc)**  **[Da]** | **Found m/z  ESI^+^ (LC-MS)** |
| --- | --- | --- | --- |
| CP‑06  (Parent peptide) | 1696.75 | 1697.85 | 849.39 (m/2) |
| CP‑06‑y1A | 1604.73 | 803.37 | 803.71 (m/2) |
| CP‑06‑S2A | 1680.75 | 841.39 | 841.72 (m/2) |
| CP‑06‑W3A | 1581.71 | 791.86 | 792.16 (m/2) |
| CP‑06‑T4A | 1666.74 | 834.38 | 834.72 (m/2) |
| CP‑06‑W5A | 1581.71 | 791.86 | 792.24 (m/2) |
| CP‑06‑G6A | 1710.77 | 856.39 | 856.41 (m/2) |
| CP‑06‑N7A | 1653.75 | 827.88 | 828.26 (m/2) |
| CP‑06‑R8A | 1611.69 | 1612.74 | 1613.70 (m/1) |
| CP‑06‑S9A | 1680.75 | 841.38 | 841.38 (m/2) |
| CP‑06‑S10A | 1680.75 | 841.38 | 842.00 (m/2) |
| CP‑06‑V11A | 1668.72 | 835.37 | 835.36 (m/2) |
| CP‑06‑R12A | 1611.69 | 1612.74 | 1613.07 (m/1) |
| CP‑06‑G13A | 1710.77 | 856.39 | 856.76 (m/2) |

Table S5: X-ray Diffraction data processing and refinement statistics for the structure of CD59 CP-06

| **Structure** | **CD59 CP-06** |
| --- | --- |
| Space Group | P2_1_ |
| Unit-cell Length (Å) | 30.94, 85.88, 96.57 |
| Unit-cell Angles | 90, 90, 90 |
| Resolution (Å) | 48.29-2.43 (2.47-2.43) |
| Unique reflections | 18920 (929) |
| Multiplicity | 3.4 (3.5) |
| R_merge_ (I) (%) | 10.2 (230.9) |
| R_meas_ (I) (%) | 12.2 (272.3) |
| *R* _p.i.m._ (I) (%) | 6.6 (143.3) |
| CC_1/2_ | 0.99 (0.29) |
| 〈*I*/σ(*I*)〉 | 7.0 (0.7) |
| Completeness (%) | 99.1 (99.7) |
| Wilson *B* factor (Å^2^) | 59.0 |
| *R* _work_/*R* _free_ (%) | 23.5/29.9 |
| R.m.s.d., bond lengths (Å) | 0.0142 |
| R.m.s.d., bond angles (°) | 2.776 |
| Maximum-likelihood-based ESU (Å) | 45.93 |
| Mean *B* Value (Å^2^) | 78.67 |
| Average *B* Value Ligands (Å^2^) | 82.32 |
| Favoured regions (%) | 95.08 |
| Allowed regions (%) | 3.52 |
| PDB code | 8CN6 |

**5. References**

1 T. E. McAllister, T.-L. Yeh, M. I. Abboud, I. K. H. Leung, E. S. Hookway, O. N. F. King, B. Bhushan, S. T. Williams, R. J. Hopkinson, M. Münzel, N. D. Loik, R. Chowdhury, U. Oppermann, T. D. W. Claridge, Y. Goto, H. Suga, C. J. Schofield and A. Kawamura, *Chem Sci*, 2018, **9**, 4569–4578.

2 Y. Goto, T. Katoh and H. Suga, *Nat Protoc*, 2011, **6**, 779–790.

3 H. Suga, Y. Goto and T. Katoh, *Protoc Exchange*. 2011 (available at https://doi.org/10.1038/protex.2011.209)

4 K. J. Leath, S. Johnson, P. Roversi, T. R. Hughes, R. A. G. Smith, L. Mackenzie, B. P. Morgan and S. M. Lea, *Acta Crystallogr Sect F Struct Biol Cryst Commun*, 2007, **63**, 648–652.

5 G. Winter, D. G. Waterman, J. M. Parkhurst, A. S. Brewster, R. J. Gildea, M. Gerstel, L. Fuentes-Montero, M. Vollmar, T. Michels-Clark, I. D. Young, N. K. Sauter and G. Evans, *Acta Crystallogr D Struct Biol*, 2018, **74**, 85–97.

6 P. R. Evans and G. N. Murshudov, *Acta Crystallogr D Biol Crystallogr*, 2013, **69**, 1204–1214.

7 P. A. Karplus and K. Diederichs, *Elsevier Ltd*, 2015, preprint, DOI: 10.1016/j.sbi.2015.07.003.

8 A. J. McCoy, R. W. Grosse-Kunstleve, P. D. Adams, M. D. Winn, L. C. Storoni and R. J. Read, *J Appl Crystallogr*, 2007, **40**, 658–674.

9 G. N. Murshudov, P. Skubák, A. A. Lebedev, N. S. Pannu, R. A. Steiner, R. A. Nicholls, M. D. Winn, F. Long and A. A. Vagin, *Acta Crystallogr D Biol Crystallogr*, 2011, **67**, 355–367.

10 P. Emsley, B. Lohkamp, W. G. Scott and K. Cowtan, *Acta Crystallogr D Biol Crystallogr*, 2010, **66**, 486–501.

11 C. J. Williams, J. J. Headd, N. W. Moriarty, M. G. Prisant, L. L. Videau, L. N. Deis, V. Verma, D. A. Keedy, B. J. Hintze, V. B. Chen, S. Jain, S. M. Lewis, W. B. Arendall, J. Snoeyink, P. D. Adams, S. C. Lovell, J. S. Richardson and D. C. Richardson, *Protein Science*, 2018, **27**, 293–315.

12 E. F. Pettersen, T. D. Goddard, C. C. Huang, E. C. Meng, G. S. Couch, T. I. Croll, J. H. Morris and T. E. Ferrin, *Protein Science*, 2021, **30**, 70–82.
